# Principles of Photocatalysts and Their Different Applications: A Review

**DOI:** 10.1007/s41061-023-00444-7

**Published:** 2023-10-31

**Authors:** Mohamed A. Hassaan, Mohamed A. El-Nemr, Marwa R. Elkatory, Safaa Ragab, Violeta-Carolina Niculescu, Ahmed El Nemr

**Affiliations:** 1 Marine Pollution Department, Environment Division, National Institute of Oceanography and Fisheries (NIOF), Kayet Bey, Elanfoushy, 21556 Alexandria Egypt; 2 Department of Chemical Engineering, Faculty of Engineering, Minia University, Minia, 61519 Egypt; 3 Advanced Technology and New Materials Research Institute, SRTA-City, New Borg El-Arab City, 21934 Alexandria Egypt; 4 National Research and Development Institute for Cryogenic and Isotopic Technologies-ICSI Rm. Valcea, 4th Uzinei Street, 240050 Valcea, Romania

**Keywords:** Doped metal oxides, CO_2_ reduction, Plasmonic nanostructures, Photocatalyst, H_2_ production, Artificial intelligence

## Abstract

Human existence and societal growth are both dependent on the availability of clean and fresh water. Photocatalysis is a type of artificial photosynthesis that uses environmentally friendly, long-lasting materials to address energy and environmental issues. There is currently a considerable demand for low-cost, high-performance wastewater treatment equipment. By changing the structure, size, and characteristics of nanomaterials, the use of nanotechnology in the field of water filtration has evolved dramatically. Semiconductor-assisted photocatalysis has recently advanced to become among the most promising techniques in the fields of sustainable energy generation and ecological cleanup. It is environmentally beneficial, cost-effective, and strictly linked to the zero waste discharge principle used in industrial effluent treatment. Owing to the reduction or removal of created unwanted byproducts, the green synthesis of photoactive nanomaterial is more beneficial than chemical synthesis approaches. Furthermore, unlike chemical synthesis methods, the green synthesis method does not require the use of expensive, dangerous, or poisonous ingredients, making it a less costly, easy, and environmental method for photocatalyst synthesis. This work focuses on distinct greener synthesis techniques utilized for the production of new photocatalysts, including metals, metal doped-metal oxides, metal oxides, and plasmonic nanostructures, including the application of artificial intelligence and machine learning to the design and selection of an innovative photocatalyst in the context of energy and environmental challenges. A brief overview of the industrial and environmental applications of photocatalysts is also presented. Finally, an overview and recommendations for future research are given to create photocatalytic systems with greatly improved stability and efficiency.

## Introduction

**Fig. 1:**
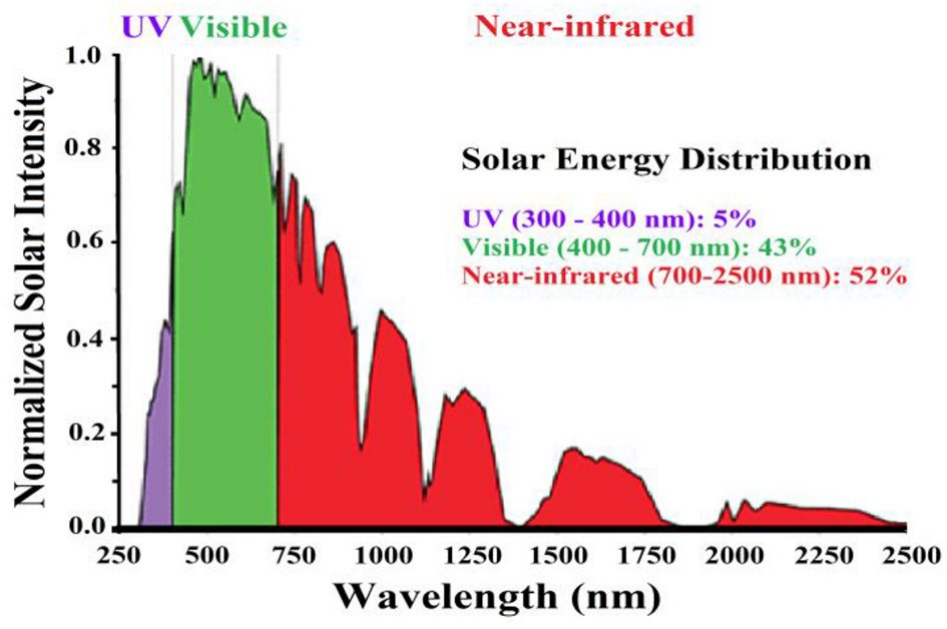
The solar energy spectrum [31]

Photocatalysts are nanoparticles (NPs) with semiconducting features such as light absorption, charge transfer, and favorable electronic structure, among others. Photoactive NPs serve as catalysts in a variety of applications, including sustainable energy production and environmental remediation [1–15]. When compared with bulk materials, photocatalysts have exceptional structures and a higher surface area to volume ratio, which boosts their actions [16, 17]. As a result, controlling the shape and size of photocatalytic materials in the nanoscale range allows for the creation and fabrication of materials appropriate for use in innovative applications. Green photoactive NPs can be made from a variety of biological sources, including plant materials and microorganisms. This synthesis approach is environmentally friendly, green, biocompatible, and cost-effective [18, 19]. Green synthesis nanophotocatalysts exhibit improved catalytic activity while reducing the usage of costly and dangerous chemicals [20, 21]. The use of diverse bacterial species for the fabrication of noble metals such as Au, Ag, Pt, Pd, and other semiconductor oxides such as TiO_2_ and ZnO is also favorable for greener synthesis of NPs. Algae, bacteria, and fungi have commonly been used in microbial-mediated processes to produce very stable metal NPs. Plant extracts are becoming increasingly popular owing to their ease of production and handling, as well as their low danger. Because light is plentiful, inexpensive, clean, sustainable, and readily available, photocatalytic pollution remediation is a potential solution. Furthermore, light can trigger highly selective reactions [22–25]. Photocatalysis can easily destroy persistent organic contaminants [26]. To enable light-induced reduction–oxidation (redox) processes that oxidize many organic contaminants by producing reactive oxygen species (ROS), new, high-performance, photoactive nanomaterials are required [24, 27–29]. For humans, sunlight is a crucial source of energy. A continual flow of electromagnetic radiation waves carries solar energy to the Earth’s surface (Fig. 1). Only a small fraction of the total energy emitted in the solar system is captured by the planet. Photosynthesis has been the most common method of converting solar energy into usable energy, which is vital to human survival via agriculture and forestry. Solar radiation has a total power of 384.6 yottawatts. A significant quantity of energy is radiated continuously by the Sun in all directions. Exposed parts of the Earth receive around 1368 W m^–2^ at a distance of 150 million kilometers [30, 31]. Because the atmosphere reflects 30% of the irradiated solar energy, about 1000 W m^−2^ of solar energy is received everywhere on the Earth’s surface. Although the total amount of energy produced by the Sun is more than enough, the true difficulty is figuring out how to collect and use it efficiently.

The importance of the synthesis of materials from natural, renewable, and biological resources will be described below. Materials such as semiconductors, noble metals, and ceramics can be made from the above-mentioned natural resources, needing little energy and having fewer byproducts to remove [32]. Carbon-based nanostructures, for example, can be made from renewable resources including sugarcane bagasse, coconut coir, peanuts, bamboo wood, groundnut shells, and coal [33]. The calcination and filtering of the above-mentioned naturally available resources use less energy to manufacture carbon-based nanostructures. In contrast to the traditional approach for generating carbon nanostructures, advanced production techniques such as chemical vapor deposition, molecular beam epitaxy, and thermal exfoliation techniques are required. Ramrez-Rico et al. [34] and Singh et al. [35, 36] found that these processes require a lot of energy, use toxic chemicals, emit dangerous gases, and have a high production cost. More items can be made in an environmentally acceptable manner without harming our society or the environment, according to the green technology vision. The use of energy and the production of harmful emissions are both reduced when using green chemistry. Nuclear, wind, and hydroelectric energy are currently available technologies for energy distribution around the world, all being produced in a more environmentally friendly manner than through the use of fossil fuels [37, 38]. Alternative energy production systems should be introduced in a greener way to balance the total requirement for energy from the world’s population. Materials are a fundamental boon to modern technology, and their performance and variety of features define the reliability and efficacy of products. Furthermore, recent progress in the development of novel materials can be used directly in energy and environmental applications. Such advanced technology can process new materials and establish a relationship between the protocol creator, material producer, designer, and developer. This is a thorough method of identifying a long-term substance for environmental activities. There will be a great global need for sophisticated materials in the energy and environmental industries in a few years. This is the only solution to avoid excessive carbon emissions and high energy consumption in energy production materials [39–41]. The current goal is to develop sustainable materials for energy and environmental-related industries; moreover, awareness of the use of sustainable materials should be promoted through education or government initiatives for the next generation. We discuss herein several greener methodologies for the fabrication of new photocatalysts. The applications of metal oxides, metal-doped metal oxides, metals, and plasmonic nanocatalysts with higher activity than bulk materials are discussed in this review. Finally, future research trends regarding photocatalytic processes are provided in detail.

## Photosynthesis and Photocatalysts as Sustainable Energy Solutions

**Fig. 2:**
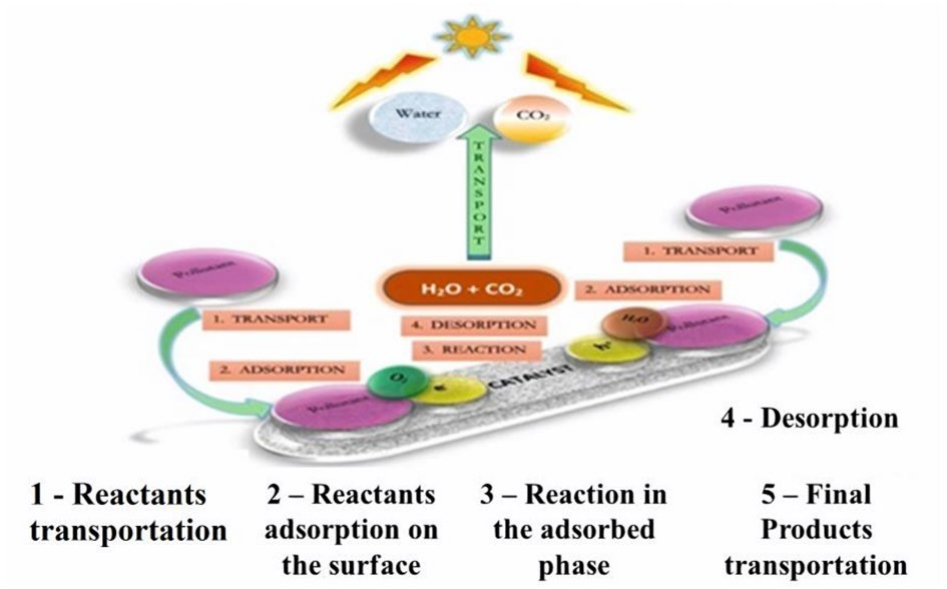
The numerous processes in a heterogeneous photocatalytic reaction, modified after Ref. [46]

Hashimoto et al. mentioned that, in the 1970s, Fujishima and Honda introduced photocatalysis, which became known as the Honda–Fujishima effect [42–44]. Water splitting (Fig. 2) is a common type of photocatalysis, in which sunlight interacts with NP catalysts (such as TiO_2_ NPs) to split H_2_O molecules into O and H atoms, resulting in H_2_ gas as an ecofriendly energy resource. Owing to its transparency to visible light, the efficiency of the water splitting process depends on nanocatalysts to collect light and transform it into chemical energy for water molecules.

The water splitting reaction can be described simply as

1 $${\mathrm{H}}_{2}\mathrm{O}+\mathrm{ light energy }\underset{ }{\to } 0.5 {\mathrm{O}}_{2}+ {\mathrm{H}}_{2}$$

Water splitting requires light with an energy of 1.23 eV, which corresponds to a wavelength of about 1 mm [45]. In theory, every photon in the visible spectrum could cause water splitting. However, water cannot absorb sunlight directly because it is transparent to the entire spectrum.

A catalyst is required to transfer photon energy to water molecules by first absorbing the sunlight and then transferring the energy to the H_2_O. Photosynthesis is the conversion of solar energy into chemical energy that can be stored in specific categories of chemical compounds. Plants break down carbon dioxide (CO_2_) and water (H_2_O) via photosynthesis to generate carbohydrates (C_6_H_12_O_6_) and oxygen (O_2_) [47–50] according to the equation

2 $${\text{6CO}}_{{2}} + {\text{12H}}_{{2}} {\text{O}} + {\text{light energy }} \to {\text{ C}}_{{6}} {\text{H}}_{{{12}}} {\text{O}}_{6} + {\text{ 6O}}_{{2}} + {\text{6H}}_{{2}} {\text{O}}$$

### Mechanism of Photocatalytic Reactions

The activation of a photocatalyst, i.e., semiconducting material, in photocatalysis is dependent on the wavelength of the radiation and the photocatalyst's “bandgap,” that is, the energy difference between the valence band (VB) and the conduction band (CB) of the photocatalyst [51–55]. When a photocatalyst such as CdS, ZnO, WO_3,_ ZnS, ZrO_2_, or TiO_2_ absorbs energy from an artificial light source or sunlight, the production of an e–h pair can occur only if the photon energy (*hυ*) is equal to or greater than the photocatalyst’s bandgap energy (*E*_g_) [55–58]. An electron in the conduction band of the semiconductor (^e−^CB) can be used to reduce any substrate, while a hole in the valence band (^h+^VB) can be used to oxidize a variety of substances. The photocatalytic reaction can take place in two ways: homogeneously or heterogeneously [59–63], but heterogeneous photocatalysis has been extensively discussed in recent years owing to its widespread application in fields such as environmental remediation, energy-related applications, and organic syntheses [59, 63].

**Fig. 3:**
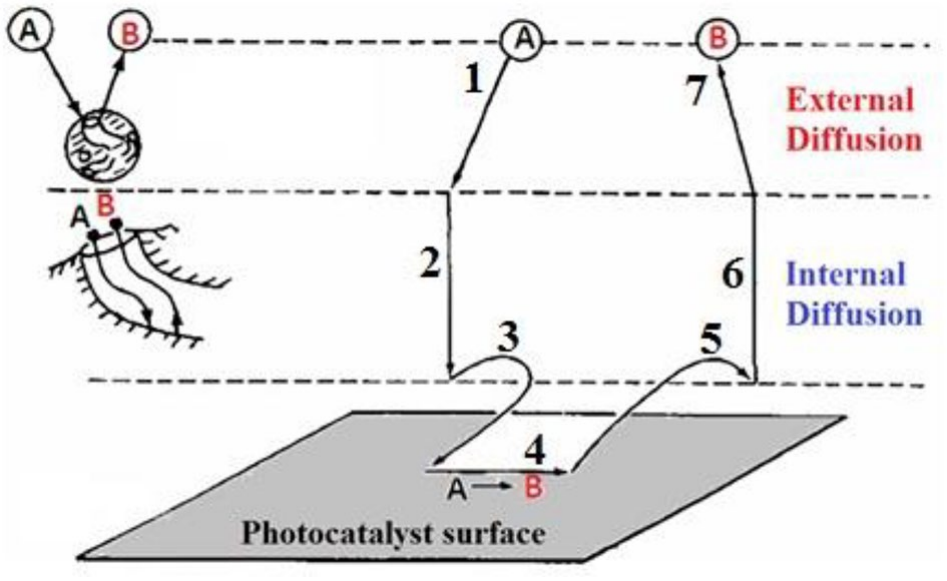
Steps involved in heterogeneous catalysis, modified after Ref. [67]

The following are the steps in the total photocatalysis reaction process [64], which are depicted in Fig. 3 [65, 66]:

After the incidence of a photon with energy (*hυ*) higher than or equal to the semiconductor’s bandgap energy (*E*_g_), the photocatalytic process (step 4) commences [68]. The following are the reactions that proceed during the oxidation of organic compounds using photocatalysts under ultraviolet (UV) irradiation [69–71]:

3 $${\text{Semiconductor photocatalyst }} + hv \to {\text{semiconductor photocatalyst}}$$

4 $${\text{Semiconductor photocatalyst }} + {\text{ H}}_{{2}} {\text{O }} \to {\text{ semiconductor photocatalyst }} + {\text{ H}}^{ + } + {\text{ OH}}$$

5 $${\text{Semiconductor photocatalyst }} + {\text{ HO}}^{-} \to {\text{ semiconductor photocatalyst }} + {\text{ OH}}$$

6 $${\text{Semiconductor photocatalyst }} + {\text{ O}}_{{2}} \to {\text{ semiconductor photocatalyst }} + {\text{ O}}_{{2}}^{{.{-}}}$$

7 $${\text{O}}_{{2}}^{. - } + {\text{ H}}_{{2}} \to {\text{HO}}_{{2}}$$

8 $${\text{HO}}^{.}_{{2}} + {\text{ HO}}^{.}_{{2}} \to {\text{ H}}_{{2}} {\text{O}}_{{2}} + {\text{ O}}_{{2}}$$

9 $${\text{Semiconductor photocatalyst }} + {\text{ H}}_{{2}} {\text{O}}_{{2}} \to {\text{ semiconductor photocatalyst }} + {\text{ OH}}^{.} + {\text{ OH}}^{ - }$$

10 $${\text{H}}_{{2}} {\text{O}}_{{2}} + {\text{ O}}_{{2}}^{{.{-}}} \to {\text{OH}}^{.} + {\text{ OH}}^{-} + {\text{ O}}_{{2}}$$

11 $${\text{H}}_{{2}} {\text{O}}_{{2}} + h\upsilon \to {\text{ 2OH}}^{.}$$

12 $${\text{Organic compound }} + {\text{ OH}}^{.} \to {\text{ degradation products}}$$

13 $${\text{Organic compound }} + {\text{ semiconductor photocatalyst }} \to {\text{ oxidation products}}$$

14 $${\text{Organic compound }} + {\text{ semiconductor photocatalyst }} \to {\text{ reduction products}}$$

**Fig. 4:**
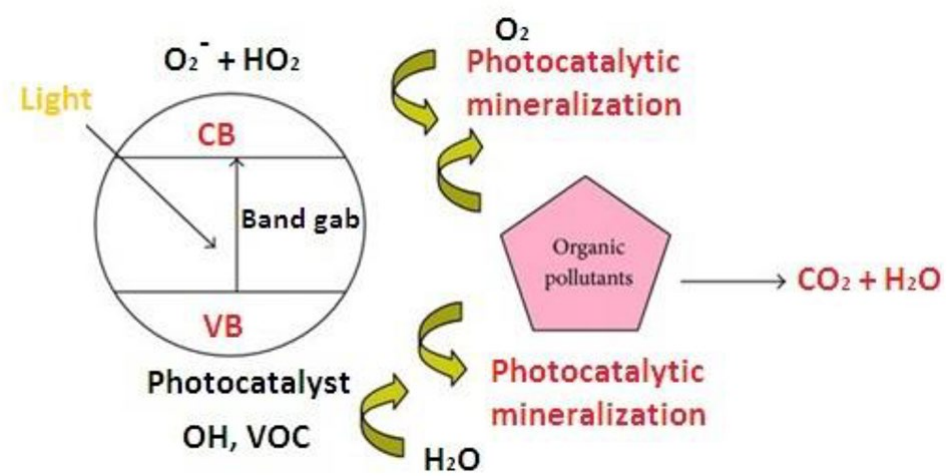
Heterogeneous photocatalysis concept for degrading organic compounds in water

When the photon energy received from the Sun or other types of artificial light sources (fluorescent lamps, light-emitting diodes (LEDs), etc.) is equal to or greater than the semiconductor photocatalyst’s bandgap energy (*h* > *E*_g_), an electron in the semiconductor photocatalyst’s occupied VB can be shifted to the unoccupied CB, resulting in an excited-state conduction-band electron (^e−^CB) and a positive valence-band hole (^h+^VB) [67]. Redox processes (Eqs. 3–14) involving numerous organic compounds adsorbed on the photocatalyst surface can easily involve electron and hole migration to the photocatalyst surface. The formation of high-energy OH occurs when positively charged holes (^h+^VB) react with surface-bound water or ^.^OH. While free radicals (Eqs. 3–4) react with oxygen to form superoxide anions, ^e−^CB reacts with oxygen to produce superoxide anions (Eq. 6). Hydroxyl radicals can also be produced by following the reaction pathway described in Eqs. 7–11. This cycle repeats until light energy becomes available. Hydroxyl radicals (^.^OH) are the primary oxidizing species in photocatalytic oxidation processes [72]. In heterogeneous photocatalysis, the oxidation paths are more important than the reduction pathways [69]. The heterogeneous photocatalytic process is depicted in full in Fig. 4.

The recombination of the photoexcited e–h pair can release energy in the form of heat. As a result, e–h pair recombination is detrimental and decreases the efficacy of the operation [70]. Furthermore, the main goal of photocatalysis is to achieve a high-efficiency photocatalytic interaction between the excited ^e−^CB and the oxidant to produce the reduced product. Furthermore, to form an oxidized component, a reaction between a positive ^h+^VB and a reductant is required. After the photocatalysis reaction, CO_2_, water, and other degradation products (such as SO_4_ and NO_3_) are formed.

### Nanostructured Photocatalysts

Numerous semiconductors have been used as photocatalysts, including metal oxides (ZrO_2_, ZnO, V_2_O_5_, TiO_2_, Fe_2_O_3_, WO_3_, and CeO_2_) and sulfides (CdS and ZnS). TiO_2_, a semiconductor photocatalyst, has gained popularity as a result of its capacity to degrade organic contaminants found in waste streams [73–75]. In aqueous conditions, this photocatalyst shows improved conformity between photocatalytic performance and stability [76]. Scientists have recently been working on developing innovative heterogeneous photocatalysts with comparatively high photocatalytic performance for the breakdown of contaminants in the presence of sunlight or UV irradiation [28, 29, 77–80]. It is worth mentioning that the synthesis of photocatalysts is critical from both an economic and practical standpoint [64].

Nanostructured photocatalysts are extremely small semiconductor particles that are only a few nanometers in size. During the last decade, the photochemistry of nanostructured photocatalysts has become a significant domain of study in physical chemistry [81]. The improved photocatalytic and photophysical capabilities of these nanostructured photocatalysts in comparison with bulk materials piqued researchers’ curiosity [82, 83]. The enhanced specific surface area and quantum size (Q-size) are primarily responsible for the aforesaid properties. The quantum size effects are caused by limited electron movement when the particle size of photocatalysts is less than a critical size limit (for example, when the particle size is reduced to the nanometer scale). Owing to the direct effect of quantum size, the semiconductor photocatalyst’s CB and VB can become discretized into energy levels. This discretization process is determined by the size of the material structure. The redox potential of the VB or the CB changes more positively or negatively as a result of discretization. The redox potential of the produced electrons and holes is increased in this way. As a result, nanostructured photocatalysts become more reactive to oxidation [15, 70, 84].

Another important consideration for catalysts is the particular surface area. The availability of additional atoms on the particular surface of the semiconductors improves the adsorption ability of nanostructured photocatalysts. The time it takes for e–h pairs to undergo interaction with the surface of semiconductor particles determines the photocatalytic efficiency of any photocatalyst. If the particle is in the nanoscale range, the diameter becomes insignificant, and the transfer of e–h pairs from the interior to the surface turns out to be very easy, increasing the rate of the redox reaction. Because the transport of e–h pairs to the surface from the interior of the catalyst is enhanced, the likelihood of e–h pair combination decreases for nanophotocatalysts. As a result, enhanced photocatalytic reactions can be accomplished, so nanostructured photocatalysts exhibit higher photocatalytic activities than bulk photocatalysts [85, 86]. A semiconductor catalyst’s photocatalytic performance is greatly dependent on its porosity distribution. The most effective catalysts have an ideal porosity distribution in the range of micro- (pore sizes < 2 nm) or mesoporous (pore sizes 2–50 nm) range. Auyeung's group published a paper on a method for regulating the structure and porosity of catalytic NPs [87]. The existence of enormous mesopores and macrosized pores increases the operative transfer of reactants for modifying the diffusional constraints during photocatalytic activities in the case of enforced nanotexture heterogeneous catalysts (such as mesoporous sieves, zeolites, etc.). As a result, both the porosity distribution and pore size play a significant role in the real-world application of well-designed nanosized porous photocatalysts [88].

## Different Approaches for Synthesis of NPs

**Fig. 5:**
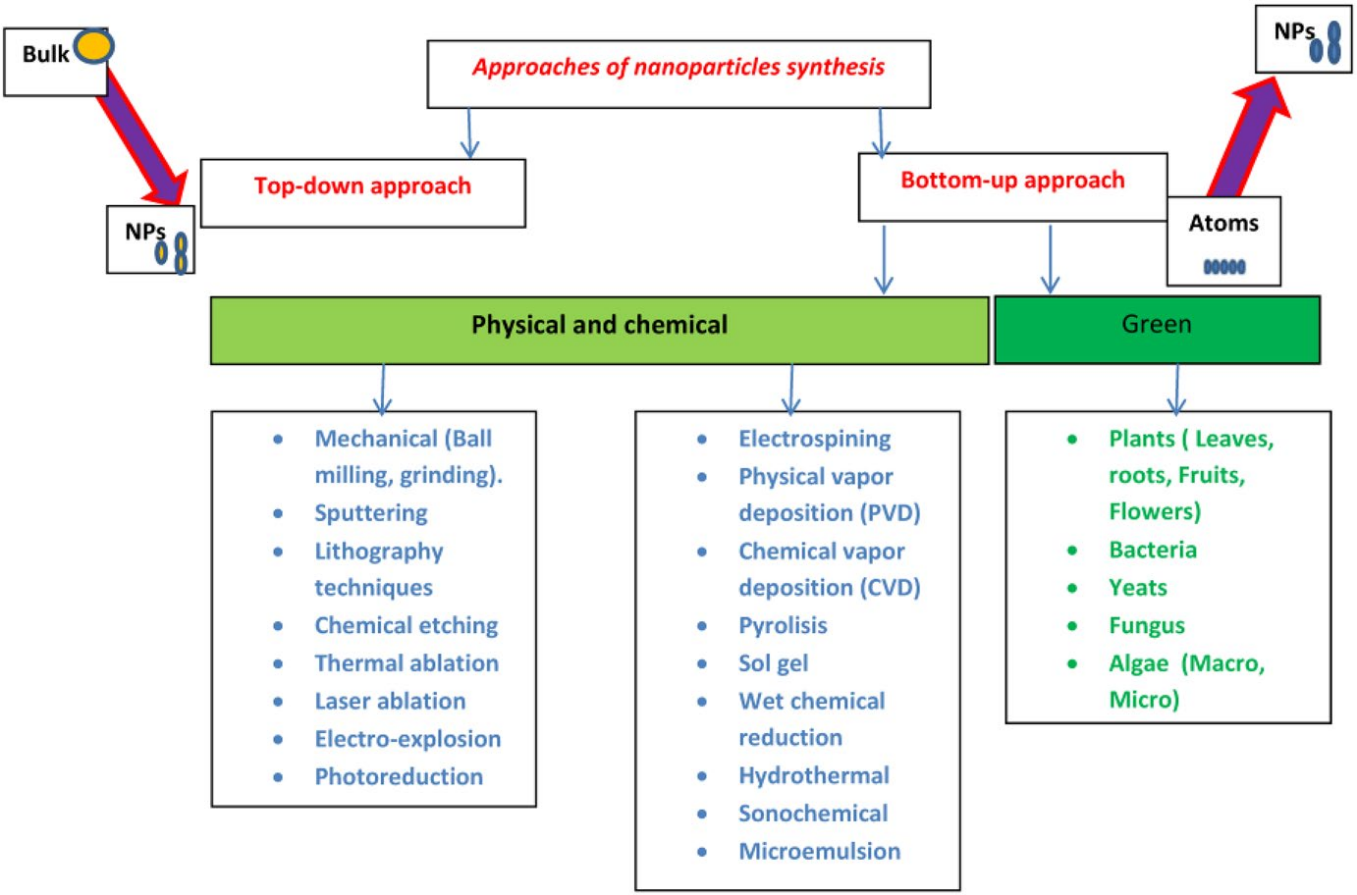
Various approaches to NP synthesis

The “top-down” technique and the “bottom-up” approach (Fig. 5) are the two basic approaches for NP synthesis. NPs are manufactured via size reduction, dissolving from bulk material into small particles, in the top-down manner [89]. Physical and chemical processes such as lithography, mechanical (e.g., milling, grinding), sputtering, chemical etching, thermal evaporation, pulsed laser ablation, and photoreduction can be used to accomplish this procedure [90–94]. The top-down technique, on the other hand, has a key flaw: the surface structure is incomplete [95]. Wet chemical methods (e.g., chemical reduction/oxidation of metal ions) and others, such as sol–gel chemistry, chemical vapor deposition (CVD), coprecipitation, microemulsion, pyrolysis, hydrothermal, solvothermal, radiation-induced, and electrodeposition methods, are used in the bottom-up approach [96–105]. Bottom-up synthesis, also known as the self-assembly technique, involves assembling NPs from smaller units such as atoms, molecules, and smaller particles [106, 107]. They are, however, disadvantageous because of the use of potentially dangerous and poisonous ingredients, high investment costs, environmental toxicity, high energy demands, long response times, and non-ecofriendly byproducts [108–110].

**Fig. 6:**
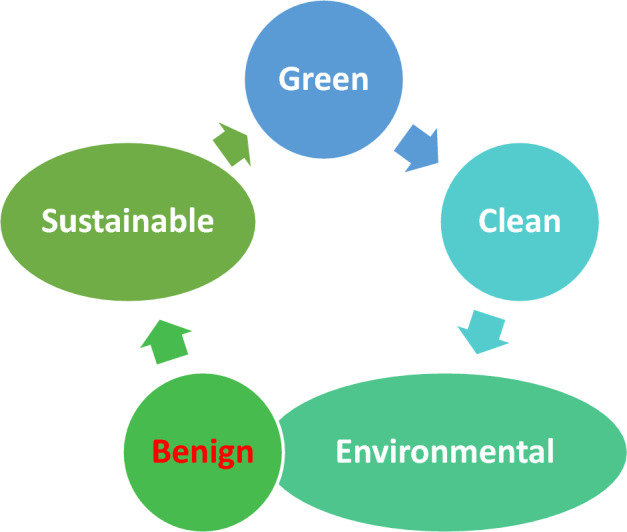
Alternate nomenclatures for environmentally friendly techniques shown in the web of green chemistry

**Fig. 7:**
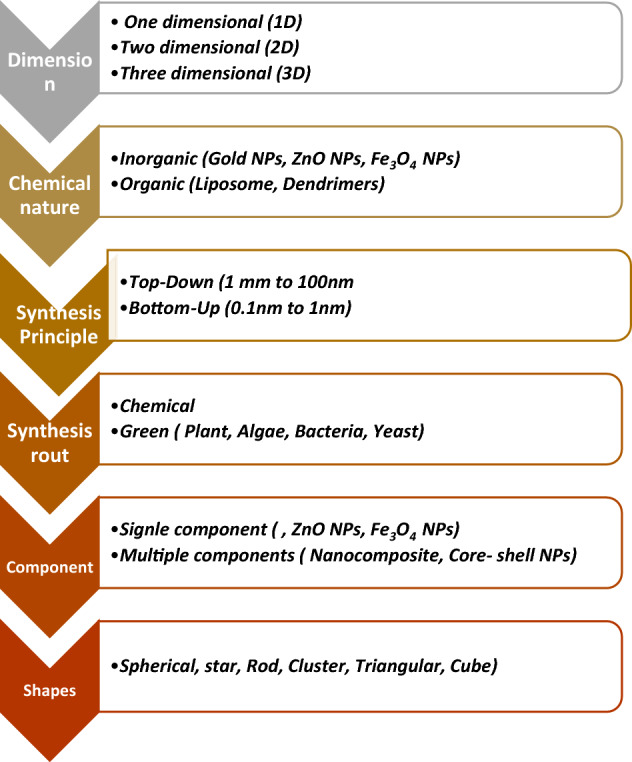
The classification of NPs through various modes

As numerous terminologies and concepts converged and diverged to build a web of green chemistry, the philosophy of green synthesis took its natural course. Figure 6 depicts the web or connectivity between various green synthesis subdisciplines. The “nano” universe is too big to be categorized into a single mode. As a result, the classification of NPs is governed by several factors, including their size, shape, content, homogeneity, and aggregation [5]. Figure 7 depicts a variety of traditional methods for the classification of NPs.

## What Is Green Synthesis?

With the publication of Rachel Carson’s *Silent Spring* in 1962, the environmental agenda gained traction, resulting in the establishment of the US Environmental Protection Agency (EPA) and the birth of green chemistry. Over time, this strategy became increasingly popular among scientists. The philosophy of green synthesis is to synthesize chemicals and molecules in an environmentally acceptable manner. NPs are no exception when it comes to using this method of synthesis.

The following are the main goals of green NPs synthesis:

• Using solvents/reagents that are safe for synthesis

• Using a low-energy conversion technique

• Using a biological process to make NPs (i.e., a biosynthetic route)

• Achieving environmentally friendly and safe NP synthesis for future applications

### How Is Synthesis Green?

As previously stated in the principles of NP synthesis via green chemistry, the main emphasis is on the environmentally friendly characteristics of the process and final product to avoid any form of hazard. Table 1 outlines a small number of attempts at manufacturing NPs that are environmentally friendly.

**Table 1:** The logic for using a green approach for synthesis of NPs

No.	NPs	Mode of synthesis	Why green?	Refs.
1	Co	Reduction of *p*-nitrophenol to *p*-aminophenol by sodium borohydride	The manufacture of air-stable NPs is made possible by a simple reduction method	[111]
2	Magnetic NPs based on Fe_3_O_4_	One-pot solvothermal method	Synthesis time is reduced by 30 min	[112]
3	Ag	Cow milk as reductant	Cow milk proteins reduce Ag^+^ ions, making it an excellent phytopathogen inhibitor	[113]
4	Ag and Au	Calcium alginate gel beads using a reductant and a stabilizer	For solid-phase biopolymer-based catalysts, a green photochemical method has been proven to be helpful	[114]
5	Au	Amine as a reducing agent and stabilizer	Using amine-reducing agents, we were able to control the creation of Au NP structures	[115]

### What Are the Approaches for Green Synthesis of NPs?

NPs can be made using a variety of techniques. However, the process can be changed with good reason to fulfill the goal of green synthesis. Table 2 summarizes a few of the green synthesis pathways.

**Table 2:** Various green synthesis methods [5]

Type	Description
*Plant*	A common approach to green nanotechnology is to use phytochemicals, carbohydrates, and biomolecules from plant extracts (Fig. 8), as reducing or capping agents for NP formation. Among all common bioreductants, plant extracts are more beneficial than other biological resources (Fig. 9). Plant-based NP production is a simple procedure that includes mixing a metal salt with a plant extract and allowing the reaction to complete at room temperature in minutes to a few hours. Properly sized NPs are created from a metallic salt solution [116]. Plant extracts can also be used to control particle size growth by modifying synthesis parameters such as the reducing agent concentration, pH, temperature, or ratio of the reactant mix
*Microbes*	Because of their genetic diversity, widespread presence, ease of access, ease of cultivation and maintenance, ease of screening, and potential for changing the structural and functional properties of NPs, microbes have proven to be an ideal choice. The technique has grown more selective and suitable as microorganisms have a variety of biocatalysts
*Solar energy*	Sunlight, the most plentiful renewable energy source on the planet, can also be utilized to mediate the synthesis of NPs, a nonbiological route to green synthesis in general. Sunlight is a great contender for green synthesis owing to its availability and environmentally benign nature
*Microwave (MW)*	Alternative energy sources are paving the way for “green chemistry,” which can significantly reduce reaction temperatures and times while reducing energy consumption. MWs are a type of electromagnetic wave consisting of pure energy radiated as a wave traveling at the speed of light. The propagation of microwaves in condensed matter is slower than in air or vacuum, where the speed of light is slower. The power and time required for production of NPs using microwaves must be regulated. Hydrothermal synthesis devices can also benefit from microwaves
*Ultrasound (US)*	Ultrasound is a nonbiological way of producing green NPs that is safe and clean. The fabrication of controllable NPs is possible. US, which has frequencies as high as 20,000 Hz, is a powerful energy carrier that allows bulk precursors to be easily broken down into NPs of a precise shape for specific applications [117]
*Mild reducing agents*	The concentration of reducing agents and stabilizing precursors has a significant impact on metal NP synthesis. This method of NP production is essentially a hydrothermal or solvothermal process. The reactant ions and/or molecules react in a compressed liquid environment, where the reactants’ dispersion is significantly superior. The presence of functional groups in the reducing or stabilizing agents has a significant impact on the NP morphology. When glucose and fructose are utilized as reducing agents, for example, changes in morphology and dispersity might be detected [118]. The reason for this is due to changes in the reaction processes of the active functional groups, which result in metal ion reduction for NP production
*Mild reaction conditions*	The synthesis process is made less dangerous and ecologically friendly by using mild reducing conditions such as ambient temperature, pH, solvent concentration, and mild surfactants as reducing agents and stabilizers. This also reduces the amount of energy required, making synthesis safer and easier

**Fig. 8:**
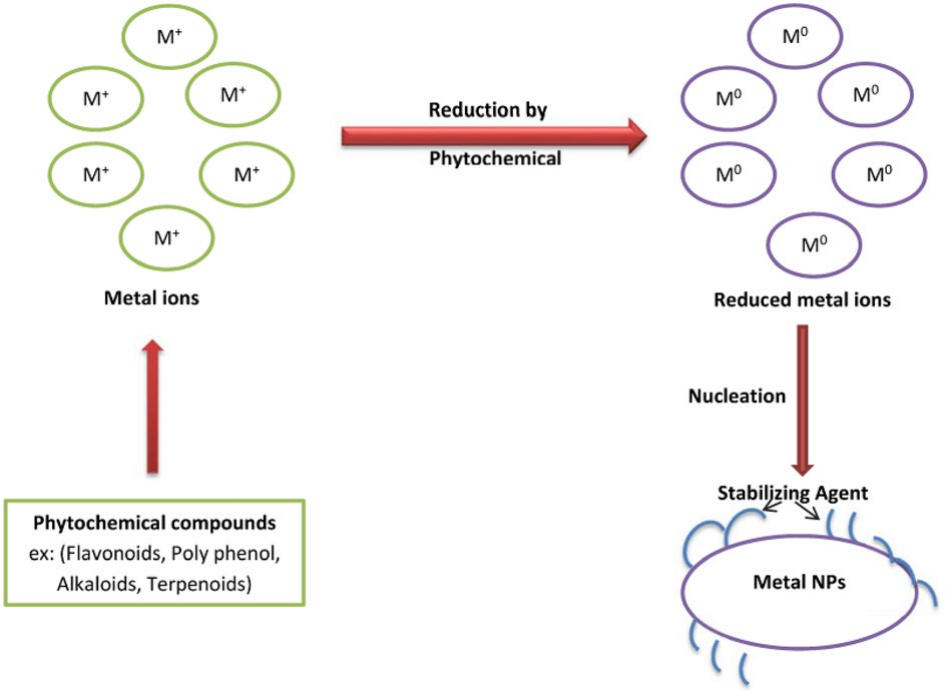
The mechanism of NP fabrication by plant leaf extract, modified after Refs. [5, 119]

**Fig. 9:**
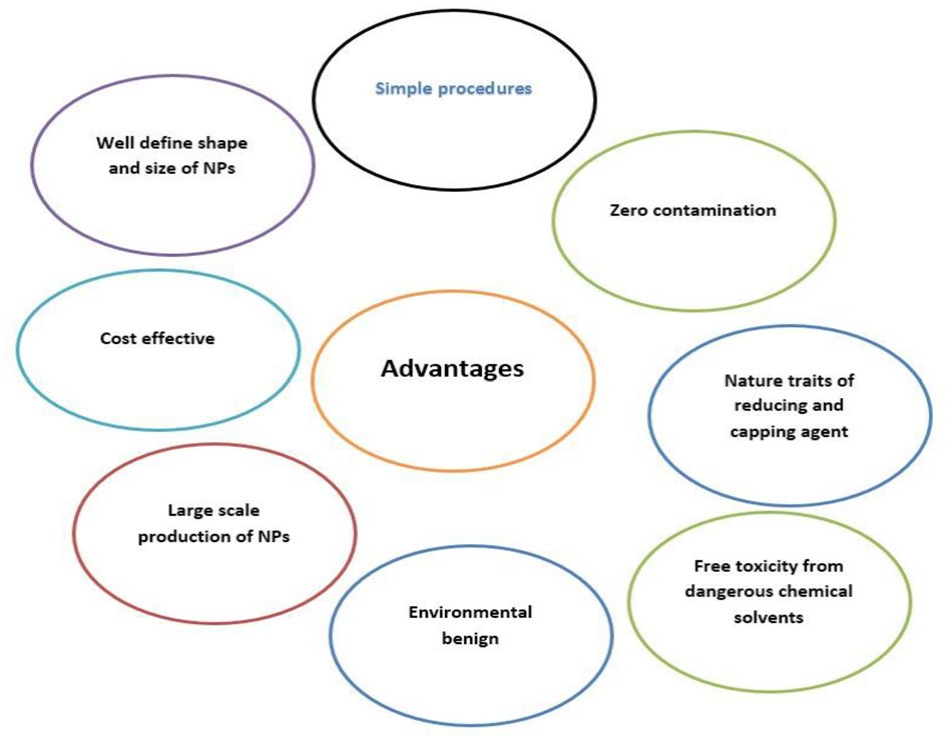
Benefits of plant-extract-mediated NP synthesis

### Green Synthesis of Different NPs and Their Applications

Figure 8 depicts several bioactive molecule compounds that have been applied in the bioreduction of metal NPs utilizing plant extracts, as well as the general bioreduction mechanisms enabled by diverse biomolecular compounds.

Alkaloids, flavonoids, saponins, steroids, terpenoids, tannins, proteins, vitamins, reducing sugars, nitrogenous bases, amino acids, and other natural phytochemical substances all contribute to metal ion reduction in plants [5, 120]. These bioactive compounds are thought to induce bioreduction in the following way:

The metal ions go through an activation phase during which they are reduced from their salt counterparts by the action of plant biomolecule metabolites with reduction capabilities, resulting in a slow development rate of particles. In the nucleation phase, new NPs are produced, and reduction processes take place as biometabolite-reducing agents, such as flavonoids or terpenoids, engage with metal ions via ionic bonding [121]. The presence of electrons as well as carbonyl groups in their molecular structure is thought to be responsible for the adsorption of biomolecule metabolite reductants on the surface of metal NPs. This is followed by a growth phase, during which the separated metal ions unite to produce metal NPs while metal ions are progressively reduced. During this process, metal ions are transformed from monovalent to divalent oxidation states to zero-valent states.

**Fig. 10:**
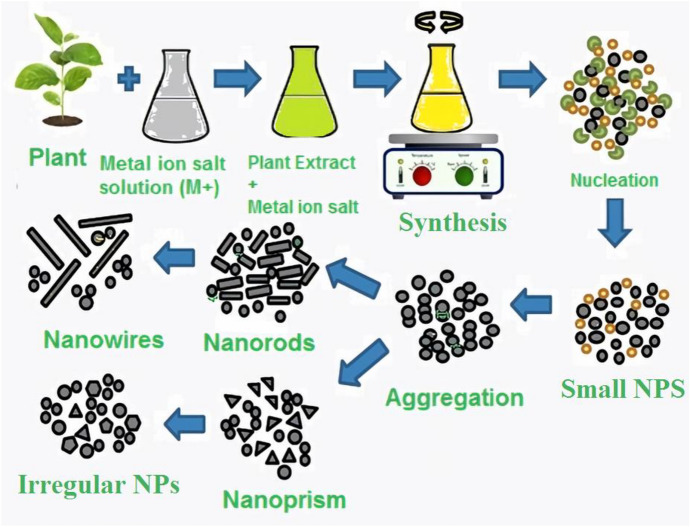
Proposed green synthesis mechanism of metal NP formation, modified after Ref. [5, 125]

NPs merge to produce a variety of morphologies as they expand, including spheres, triangles, hexagons, pentagons, rods, wires, and cubes [122]. The longer nucleation stage may result in aggregation of the resulting NPs, altering their morphologies, whereas the continuous growth stage leads to increased thermodynamic stability of NPs. Plant metabolites crown the final step of the termination phase, in which the NPs achieve their most actively helpful and stable form [123, 124]. This mechanism, together with the synergistic adsorption of functional groups from plant extract components, will create steric repulsion, limiting NP aggregation. Figure 10 shows a more detailed depiction of this potential procedure.

### Green Photocatalysis

Fujishima and Honda described the process of splitting water into hydrogen and oxygen with the use of metal oxide photocatalysts under UV light for the first time in 1972. This work has since been expanded beneficially, yielding major efficiency gains [126]. Owing to the significant potential in the realm of energy and environmental challenges, this field has piqued the interest of academics since 1972. Metal oxide semiconductors have played an important role in photocatalysis for decades owing to their high reactivity, stability, chemical inertness, and low cost. TiO_2_, ZnO, MoO_3_, Fe_3_O_4_, and other metal oxide semiconductors are ideal materials for water remediation, hydrogen fuel production, removal of toxic waste from water bodies, and pharmaceutical waste removal applications [56, 127–129]. These materials have outstanding physicochemical, electronic, and structural properties that enhance their performance in the applications stated. The process of transforming solar or light energy into chemical energy is known as photocatalysis. The terms “photo” and “catalysis” both refer to the process of speeding up, breaking down, or degrading a chemical reaction. Overall, photocatalysis helps to degrade harmful contaminants in aqueous solution by breaking them down with the use of light energy and successfully activating chemical reactions [130].

Photocatalysts can be divided into various categories, with modern research focusing on three of them: semiconductor photocatalysts, plasmonic photocatalysts, and heterogeneous photocatalysts. The following sections go over the results of a detailed survey of each kind. In general, green photocatalysis is a synthetic approach that aids in the preparation of various photocatalysts using natural resources, biomasses, and biological extracts [131]. Those precursors (resource materials) are environmentally friendly, green sources, offering economically viable routes for converting light energy to chemical energy over semiconducting materials, such as removing pollutants from water bodies, reducing toxic molecules, and producing hydrogen fuel [131–133].

#### Sunlight-Driven Photocatalysts

One of the most significant problems in the discipline of materials science is identifying acceptable metal oxide semiconductors for use as photocatalysts for the treatment of pollutants in water systems utilizing solar energy. A perfect photocatalytic material would have the required bandgap qualities to absorb a broad range of the solar spectrum, dissociate water molecules, and remain stable in a water environment during reaction processes. It should also be cost-effective, simple to process, readily available, and nontoxic to the environment. Various metal oxide semiconducting-based nano-assemblies have been created and proven to act as catalytic materials for water remediation under sunlight throughout the past few decades. The transition or d-block metal ions have shown excellent efficiency in semiconductors that have been extensively investigated as effective photocatalytic materials [28, 29].

The following factors affect the photocatalyst process:

(a) **Dye concentration**: A significant aspect of the photocatalytic reaction is the dye concentration. The catalyst should be capable of degrading a reasonable amount of dye. A small amount of dye is adsorbed on the catalyst’s surface, which causes a photocatalytic reaction in light-stimulated conditions. The adsorption of dye on the photocatalyst surface is proportional to the dye concentration at the start. The initial dye concentration is an important parameter that should be closely checked. The proportion of dye degradation reduces with increasing dye concentration, but the needed quantity of photocatalyst must be conserved [134].

(b) **Catalyst amount**: The amount of catalyst used in the photocatalytic reaction has an impact on dye degradation. In a heterogeneous photocatalytic process, one can increase the amount of photocatalyst in the reaction process to raise the proportion of dye photodegradation. More active sites are created in the photocatalytic reaction by increasing the catalyst number, which results in more reactive radicals being formed during photodegradation [135].

(c) **pH**: The pH of the solution is also important in the degradation process. Depending on the nature of the material and the qualities of the pollutant, the photocatalytic reaction can be either induced or suppressed by it. The surface potential of the catalyst (metal oxide NPs) can be altered by changing the pH of the solution. Pollutant adsorption on the photocatalyst surface may be affected as a result, causing an alteration in the photodegradation rate [136].

(d) **Surface morphology of the photocatalyst**: Significant parameters to be examined for photodegradation activity, such as particle size and shape, are included in the surface morphology. Each morphology is the result of a direct interaction between the catalyst’s surface and the organic contaminant [137]. The quantity of photons striking the photocatalyst’s surface can regulate the rate of photocatalytic activity. If the photocatalyst exhibits a range of morphologies, the reaction proceeds more quickly [138].

(e) **Surface area**: Materials with larger surface area should be used to achieve higher photocatalytic performance. Many active sites can be generated on the photocatalyst surface using these materials, resulting in the production of more radical reactive species for effective photodegradation [139].

(f) **Temperature-dependent reaction**: The temperature of the reaction should fall within this range of 0–80 °C to achieve efficient photocatalytic activity. When the temperature rises over 80 °C, the catalyst promotes e–h pair recombination and suppresses photocatalytic activity. As a result, the reaction temperature is critical for the photocatalytic activity [140, 141].

(g) **Nature of the pollutants and their concentrations**: The number and composition of specific contaminants in a water matrix can impact the degree of photodegradation. When the pollutant concentration is higher, toxic pollutants cannot be addressed by photocatalysts such as TiO_2_ as this saturates the photocatalyst surface and prevents the formation of active radicals, lowering the photocatalytic effectiveness [142].

(h) **Irradiation period and intensity of the light**: The incoming light intensity and irradiation period are important parameters in pollutant photodegradation. The photodegradation percentage is inversely related to the intensity of light at high light intensities owing to the production of excitons and the sluggish recombination of e–h pairs. Alternatively, the photocatalytic surfaces undergo e–h pair recombination, which decreases the reaction medium’s catalytic activity when the light intensity is increased [143].

i. **Dopants on dye degradation**: TiO_2_ NPs that absorb photons at very low energies can be made using a variety of ways. Bandgap engineering entails the insertion of metals and nonmetals into photocatalytic materials to continuously alter and move the VB and CB. Surface modification can be accomplished by combining organic and semiconductor materials [144].

#### Metal Oxides

Photocatalysis using semiconducting metal oxide-based nanostructures has been applied to clean wastewater and manufacture hydrogen fuel by splitting oxygen and hydrogen, among other things. The most important qualities of a photocatalytic material are its bandgap, optimum band-edge position, large surface area, perfect morphology, chemical stability, and reusability. TiO_2_, ZnO, SnO_2_, Cu_2_O, and WO_3_ with these parameters have identical photocatalytic properties, such as light absorption, among diverse metal oxide semiconductors. This stimulates photogenerated charge carriers, resulting in the formation of holes that are capable of oxidizing organic compounds [145]. Direct sunlight, visible light, ultraviolet (UV) light, or a combination of both are used to activate semiconducting metal oxide nanostructures in this reaction. The e–h pairs are formed when photons excite charge carriers from the VB to the CB. The oxidation and reduction reactions that break down the molecular chains of organic contaminants use these photogenerated e–h pairs. The semiconducting metal oxide’s photocatalytic activity is based on two broad features [146]: (i) the oxidation of OH^−^ anions to produce hydroxyl radicals, and (ii) the reduction of O_2_ to produce superoxide radicals. These radical reactive species can disinfect or mineralize organic contaminants into harmless byproducts. As a result, this process has enormous scientific significance in the fields of the environment, hydrogen fuel production, and energy. Photocatalytic materials are commonly used to remediate wastewater by removing pathogens and other hazardous contaminants.

**Fig. 11:**
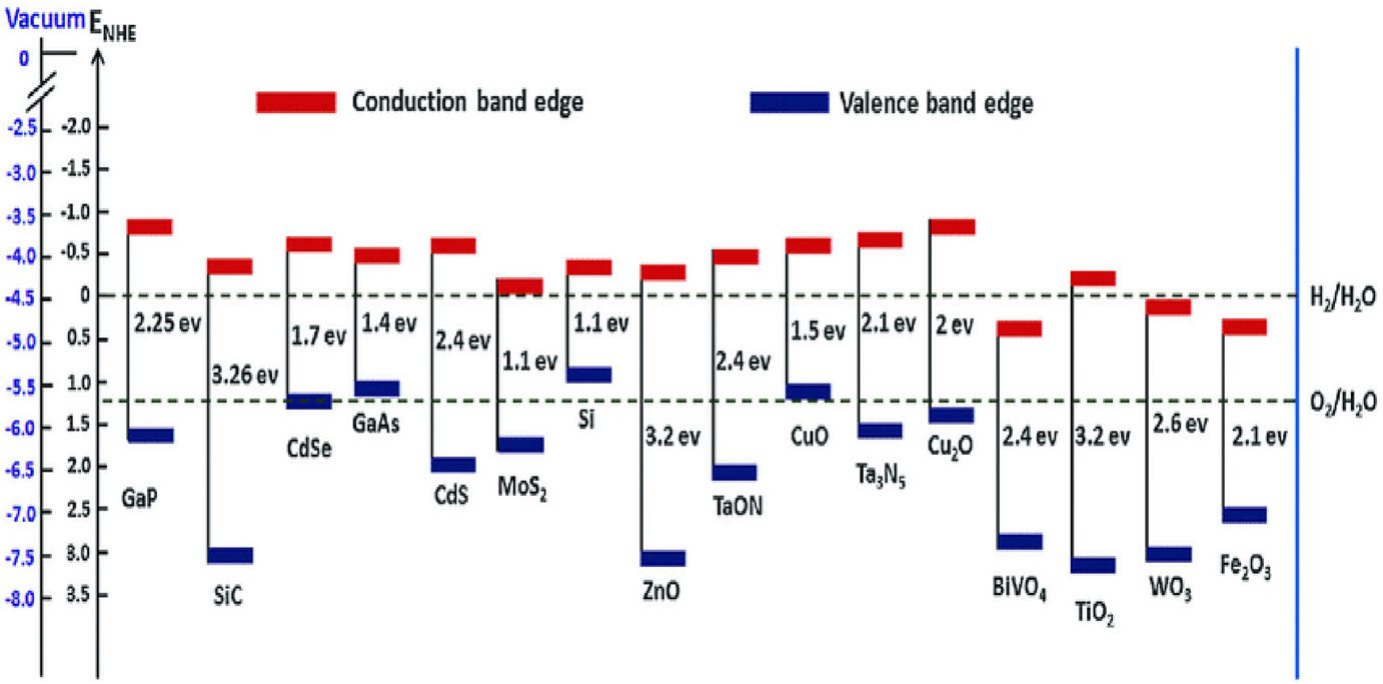
Band-edge position of different metal oxide semiconductors in comparison with a standard hydrogen electrode [150]

The bandgaps and band-edge positions of various commonly used photocatalytic semiconducting materials are shown in Fig. 11 [147]. Although some of these semiconducting materials, such as ferric oxide (Fe_2_O_3_), have sufficient bandgap energies to act in the visible light area, their use as efficient photocatalysts suffers from certain disadvantages. Researchers are looking for alternative materials to solve these problems. Metal chalcogenide semiconductors (e.g., PbS and CdS) have been shown to exhibit photocatalytic activity. However, these show limited stability and hazardous effects, and are prone to photocorrosion. Meanwhile, metal oxide semiconductors with edges of the CB that fall below the normal hydrogen electrode (NHE) potential, such as SnO_2_, WO_3_, and Fe_2_O_3_ exhibit excellent stability and photocorrosive characteristics in aqueous solution. The charge transfer property for hydrogen evolution during water splitting is initiated by using an external voltage, according to Gupta et al. [148]. When compared with TiO_2_ and ZnO materials, Fox et al. found that Fe_2_O_3_ showed poorer photoactivity owing to corrosion or the creation of short-lived charge transfer states between metal and ligand [131]. Bahnemann et al. [149] fabricated a ZnO material and discovered that it dissolves in water over time because of its deteriorating stability. TiO_2_ nanomaterial, on the other hand, is corrosion resistant and shows excellent aqueous stability.

#### Metal-Doped Metal Oxides

**Fig. 12:**
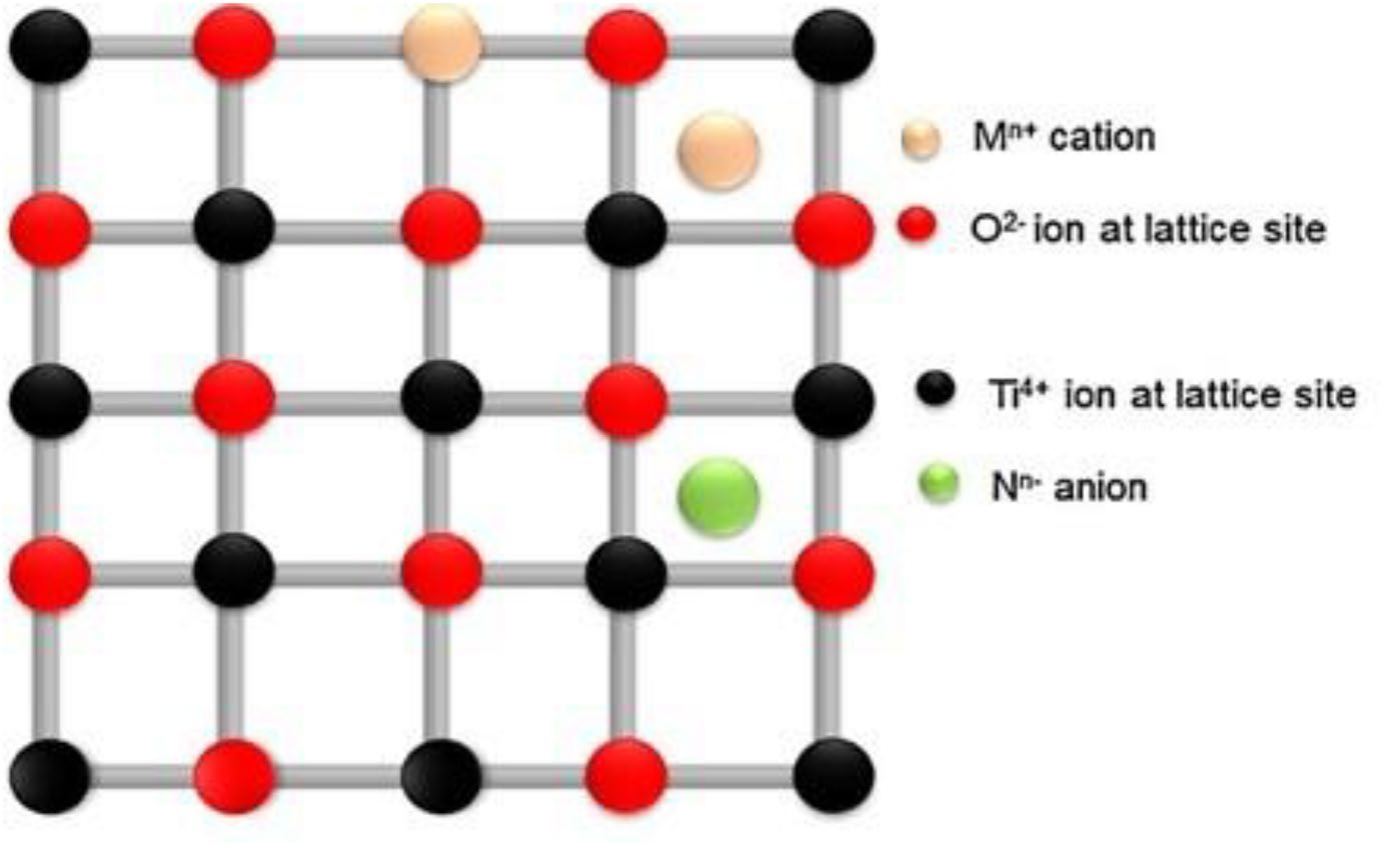
Two types of doping depicted in the lattice structure of TiO_2_ crystals: substitutional and interstitial doping, modified after Ref. [147]

Doping is the introduction of impurity atoms into any semiconducting material’s lattice system, as stated by Neamen [151]. The properties of the host material are influenced and engineered by the dopant atoms in the semiconductor lattices. A graphical example of faults in a lattice structure is shown in Fig. 12. Replacement or substitutional doping refers to substituting any impurity or foreign atom for one or more host atom. The following conditions must be met: (i) the crystal structure, electronegativity, and solubility states of both the host metal and the dopant metal must be the same, and (ii) the difference in atomic radii of the dopant atoms must not exceed 15%.

**Fig. 13:**
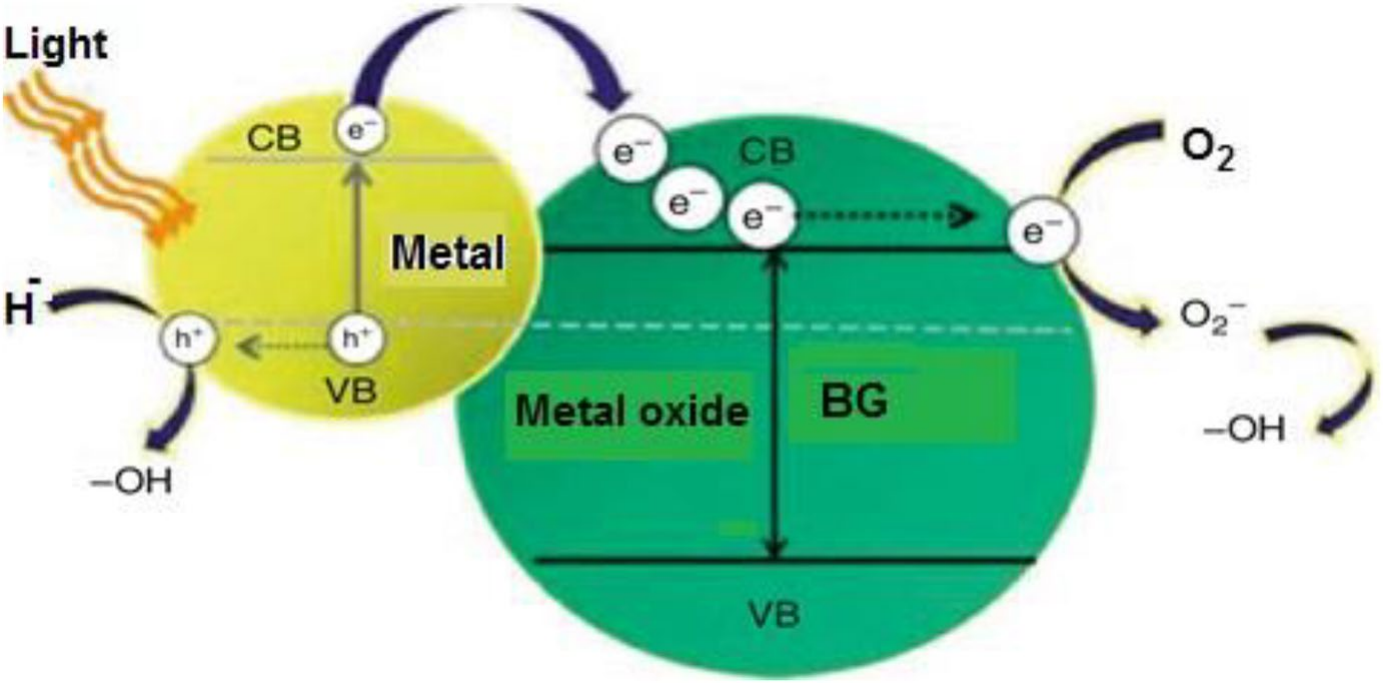
A semiconducting photocatalyst doped with metal NPs, modified after Ref. [153]

This type of doping is known as interstitial doping because the foreign atoms are wedged between typical lattice positions. The atoms are pushed out of the lattice, leaving voids between the host atoms. The interstitial and host atom radii can be evaluated to determine the chances of atoms entering interstitial locations. The atomic radius variations determine the precise location of the dopant atoms in the interstitial sites. Such cation/anion ratio (*r*^+^/*r*^−^) ionic radius measurements can be used to determine which cations will be present in certain interstitial locations. Cation coordination numbers at interstitial sites are determined by the ionic radius ratio, such as 6 (octahedral), 4 (tetrahedral), and so on. When the ionic radius ratio is increased, the number of anions surrounding the cations increases as well. Cations are wedged between anion planes in the tetrahedral holes of the tightly packed structures when the ionic radius ratio is between 0.225 and 0.414. If it lies between 0.414 and 0.732, they are filled with octahedral holes [151]. Metal or nonmetal doping or the doping of molecules with semiconductors appropriate for photocatalysis is the best strategy for increasing a photocatalyst’s absorption capabilities and modifying its electrical properties (Fig. 13). All of these factors, as well as the dopant’s surface chemical composition and ionic radius, can influence the efficacy of the doping process. Metal and nonmetal doping have received a lot of attention in recent years. Many studies have examined the use of nonmetals (e.g., boron, sulfur, and carbon) as dopants in semiconductors to change the bandgap and band-edge position of a semiconductor to make it a visible-light active photocatalyst.

Because of their greater bandgap energy, photocatalysts with semiconducting surfaces, such as TiO_2_, SnO_2_, and ZnO, can be made to operate in the ultraviolet range. It helps to change the semiconductor’s electrical structure and adjust the band-edge positions by moving the absorption range of the semiconductors into the visible light area by doping with suitable metal or nonmetal ions. Under visible light irradiation, the doped semiconductor demonstrates exceptional photocatalytic performance [152]. Recent decades have seen much research into the active photodegradation of hazardous organic pollutants, pharmaceutical waste, and deadly colors by using metal-doped TiO_2_ NPs under visible light. The absorption of TiO_2_ in the visible spectrum may be affected by structural variations. Furthermore, since the early 1990s, extensive research has been done on TiO_2_ NPs doped with nonmetals such as nitrogen, carbon, fluorine, and sulfur. These photocatalysts have demonstrated exceptional activity against a variety of contaminants when exposed to sunlight.

#### Plasmonic Photocatalysts

Plasmonic photocatalysts have piqued researchers’ interest because of their improved performance under visible light irradiation, wide spectrum of sunlight absorption, and better charge transport abilities [26, 154–157]. This type of material architecture can be formed by scattering noble-metal NPs on top of a semiconductor. Two separate characteristics are achieved in this manner: localized surface plasmon resonance (LSPR) and a Schottky barrier [26, 158]. Under visible light irradiation, these characteristics will aid in the efficient separation and transfer of charge carriers in the presence of visible light. The LSPR, which denotes significant oscillation on the surface of metal NPs and semiconductor photocatalysts, is the most important property for plasmonic photocatalysis.

**Fig. 14:**
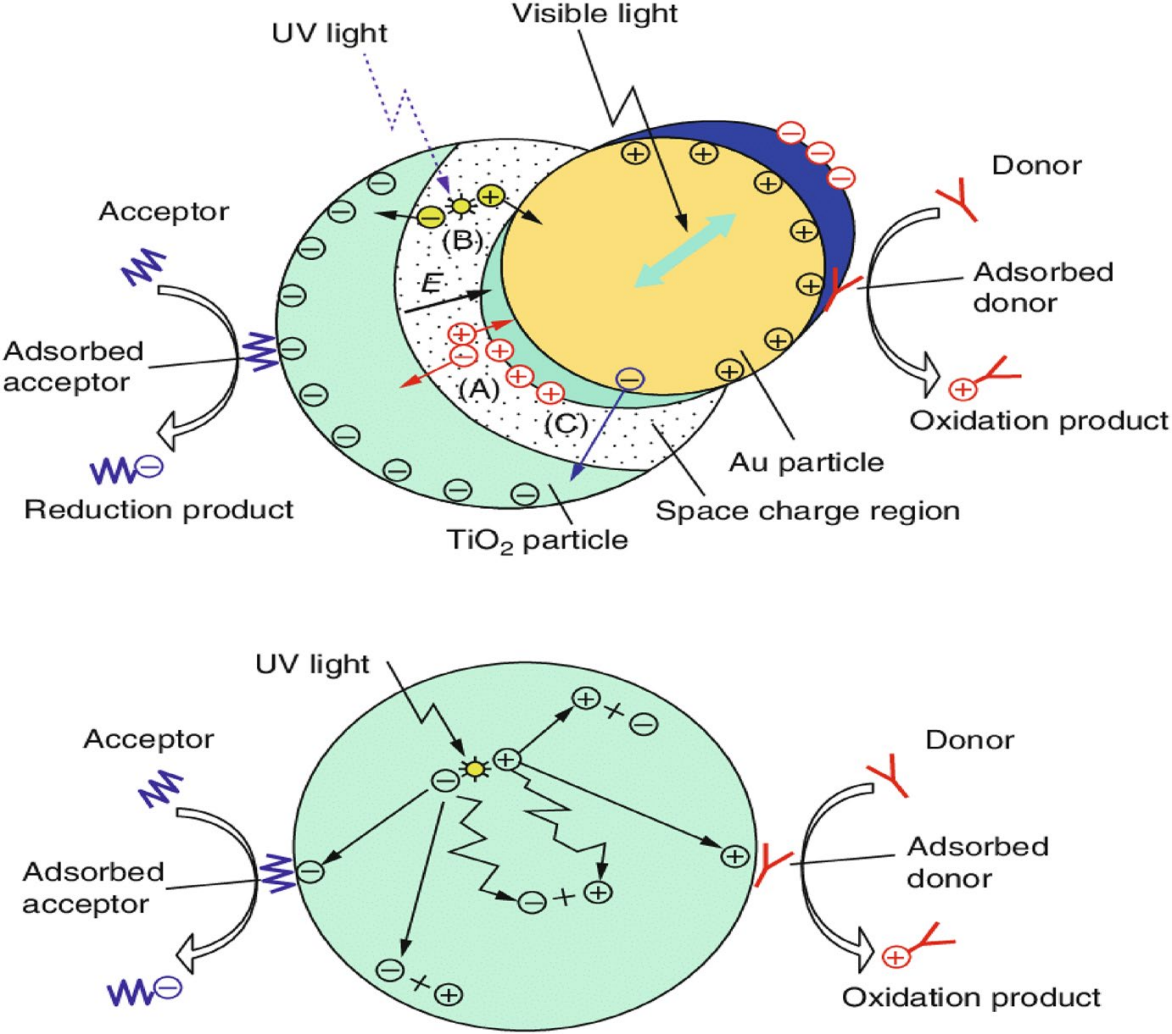
Comparison of plasmonic photocatalysts with semiconductor photocatalysts, illustrating the charge transfer mechanism [26]

Because of its short diffusion length and the interfacial charge transfer effect at the heterojunction, the metal–semiconductor junction in plasmonic photocatalysts aids in efficient e–h separation and allows charge carriers to be transferred quickly. Metal NPs such as Ag, Au, and Pt exhibit resonance oscillations at specific wavelengths that depend on the NP morphology, shape, and size. Plasmonic metal NPs exhibit resonance oscillations, which can change the absorption range of a UV light-active photocatalyst (such as TiO_2_) into the visible range. The surface plasmon resonance phenomenon in metal NPs can dramatically boost the visible-light absorption capabilities of a low-bandgap semiconducting photocatalyst such as Fe_2_O_3_. In a very thin layer of metal NPs, the total incoming light absorption can significantly improve the electron transport capabilities of a semiconductor with weak electron transport qualities. The distance between the photogenerated e–h and the noble-metal NP surface is tiny, as is the diffusion length. The charge carriers’ transport characteristics are thus improved when photons are excited [26, 159]. The most significant mechanisms involved in plasmonic photocatalysis are depicted in Fig. 14. Au NPs are partially ringed on the TiO_2_ photocatalyst surface. Because of the material’s excess electrons and defects in the native oxygen, TiO_2_ NPs have *n*-type properties in general [160]. Conventional TiO_2_ photocatalysts have been compared and analyzed thoroughly to assess the photocatalytic efficacy of Au–TiO_2_ nanostructures. Figure 14 shows how NPs of Au on the TiO_2_ surface can absorb all wavelengths of electromagnetic radiation in the visible spectrum, according to LSPR. TiO_2_ e–h collective oscillation uses an interfacial charge transfer technique to aid in the reduction and oxidation of hazardous contaminants in an aqueous medium.

When compared with typical TiO_2_ photocatalysts, this interfacial charge transfer action significantly reduces electron and hole recombination. The recombination of electron–hole pairs is a significant factor that can alter the semiconductor’s photocatalytic efficacy. One of Au's most important functions in plasmonic photocatalysts is to make it easier to absorb visible light and to prevent electron–hole pair recombination. As a result, the material may be capable of higher photocatalytic efficiency than traditional TiO_2_ photocatalysts.

#### Carbon Family

Carbon nanomaterials have recently received a lot of attention owing to their unique physicochemical, structural, optical, and electrical capabilities [161]. A wide range of nanocomposites can be created for use as conventional and traditional photocatalytic materials for light-derived water remediation applications while retaining the advantageous properties of carbon materials [162]. Water disinfection, oil adsorption, and pathogen removal from water bodies have all been investigated using nanomaterials in various forms. The physicochemical features of carbon-based nanostructures, for example, can remove organic, inorganic, and other heavy metal contaminants from water sources. Carbon-based adsorbents are commercially available. Because of their quick photodegradation and charge transport capabilities, fullerenes, graphene, and carbon nanotubes are currently utilized as cocatalysts for traditional photocatalytic materials such as TiO_2_, ZnO, SnO_2_, and others. Above all, carbon-based nanomaterials have the potential to break down complex and dangerous contaminants in water bodies with high efficiency. Under UV light illumination, graphene-decorated TiO_2_ nanocomposites have demonstrated high photocatalytic activity, according to Kamat et al. [163]. This nanocomposite breakthrough was demonstrated in depth using numerous material combinations for improved activity. As a result of their excellent physicochemical properties, strong electron-accepting capacity, regulation of work function, and electronic characteristics, graphene and its derivatives outperform other carbon nanostructures in catalysts against a variety of contaminants. These characteristics make graphene-based nanocomposites great candidates for photocatalytic applications, as they can modify the photocatalytic performance of semiconductors. Suárez-Iglesias et al. used a variety of photocatalysts to generate variations in morphology, joining with graphene via electrostatic interaction or chemical bonding for photocatalytic applications. Organic, inorganic, a combination of metalorganic frameworks, semiconductors, plasmonic metals, nonmetal plasmonic materials, and dyes are all employed as photocatalysts [164].

Catalytic materials such as graphite, activated carbon, and soot have long been replaced by carbon nanotubes (CNTs) because of their superior catalytic properties [162, 165]. Because CNTs have many active sites and a large surface area, they have recently been discovered to be effective at absorbing some hazardous chemicals. When compared with pristine CNTs and pristine TiO_2_, the integration of TiO_2_ into the CNT matrix has proved to result in an effective photocatalytic material with increased activity. The valence band traps electrons, allowing a broad range of visible light to be absorbed. The recombination rate of e–h pairs may be greatly reduced by using these nanocomposites. The energy gap of the composite nanostructures can be reduced to achieve visible light absorption of the nanocomposite.

Organic materials provide additional energy and environmental advantages, such as chemical inertness, affordability, and the capability to tackle a wide range of environmental challenges. Metal–organic frameworks, graphitic carbon nitride, and various organic dyes can be linked together with graphene or CNT nanostructures, metal–organic frameworks, graphitic carbon nitride, and other organic dyes to be employed as photocatalysts [166]. During the coupling, a Schottky-type heterojunction is formed between the organic semiconductor and graphene surfaces, which efficiently facilitates charge transport throughout the junction. The structural, electrical, and physicochemical features of graphene–organic semiconductors are facilitated by this type of layered design with metal-free photocatalysts, resulting in remarkable photocatalytic performance. Using graphene/g-C_3_N_4_ nanocomposites, Xiang et al. demonstrated the use of a combination of chemical reduction and impregnation techniques. The authors claimed that, by splitting water into hydrogen and oxygen, this material might generate hydrogen.

#### Z-Scheme in Photocatalysis

**Fig. 15:**
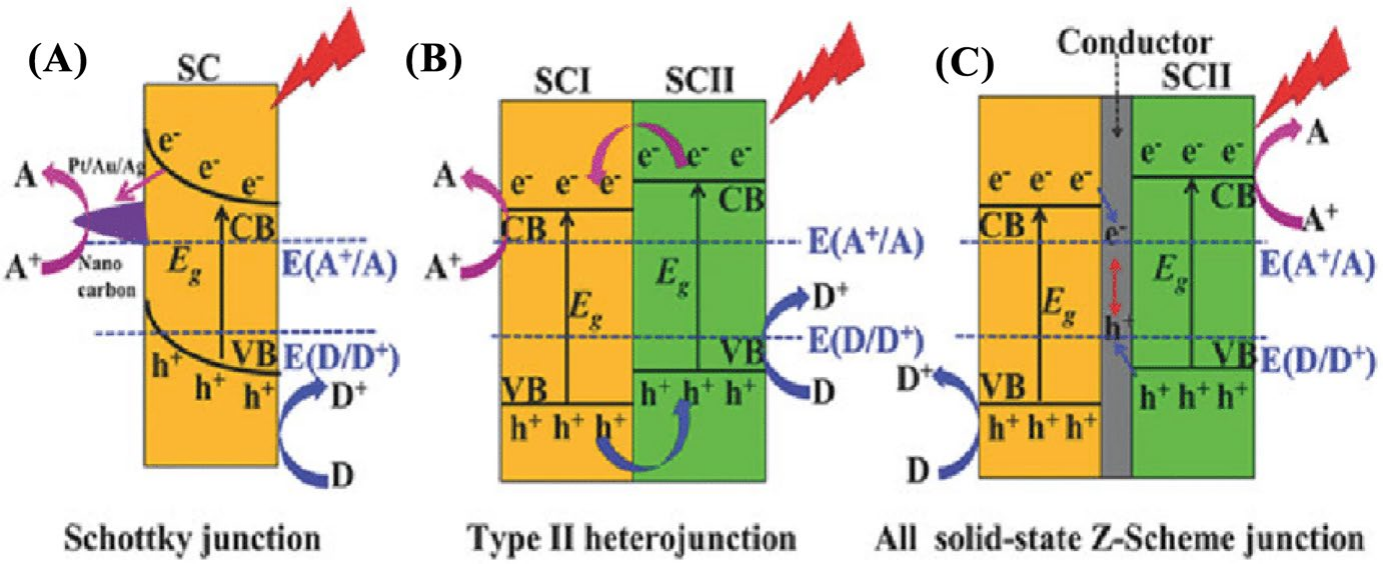
Three alternative kinds of photocatalysis system, illustrated with a band-edge diagram [166]

Because they are capable of capturing the visible spectrum of electromagnetic radiation and causing multiple photodegradation reactions, the design and development of properly assembled metal oxide-based semiconducting photocatalysts are encouraging the use of nanomaterials to address environmental issues. In photocatalysis, the development of the Z-scheme has many advantages, including excellent sunlight harvesting capability, a high degree of redox competency, as well as the ability to quickly create active species for oxidation and reduction processes, all of which contribute to improved photocatalytic activity (Fig. 15). In such a photocatalysis system, two semiconducting photocatalyst materials are connected by an appropriate redox mediator in the Z-scheme. This technology is more efficient at utilizing or absorbing sunlight than a traditional photocatalysis system. In addition, the quantity of energy required to activate the Z-scheme is reduced. Any of the photocatalysts in this system can be used as a water oxidation and reduction potential dual-purpose system. Tada et al. described a method for making CdS–Au–TiO_2_ nanocomposites and how charge transfer processes affect the photocatalytic activity. In more detail, Au NPs can be placed between CdS and TiO_2_ NPs, influencing the TiO_2_ photoinduced electron transfer and CdS NPs photoinduced hole transfer. Thereby, the photoinduced electrons have a strong reduction ability on CdS, while photoexcited holes have a significant oxidation ability on TiO_2_ NPs. A shuttle redox mediator system is another name for this electron transport mechanism. Only Z-scheme photocatalysts benefit from this type of charge transfer mechanism. These two semiconducting systems are known as Z-scheme photocatalysts because the charge transfer processes in them resemble the shape of the letter “Z” [63, 167–170].

## Green Materials for Green Photocatalysts

### Green Synthesis of Novel Photocatalysts by Microorganisms (Bacteria, Fungi, and Algae Biosynthesis) (Table 3)

**Table 3:** Different biobased sources for photocatalyst nanoparticle synthesis and their different applications

Extract type	Photocatalyst	Size	Shape or application	Refs.
*Fusarium oxysporum*	ZnS	42	Spherical	[200]
*Fusarium oxysporum*	CdS	5–20		[201]
*Coriolus versicolor*	CdS	8–15	Globular	[202]
*Phanerochaete chrysosporium*	CdS	1.5–2	Spherical	[203]
*Calotropis gigantean*	CdS	20	86% MB and 91% EY removal	[204]
*Citrus limetta*	ZnS	27	83% RhB removal	[205]
*Corymbia citriodora*	ZnS	45	96% MB removal	[206]
*Ficus johannis*	ZnS	1.4	> 90% Naph, AnT, 4-CP, 4-NP removal	[207]
*Dicliptera roxburghiana*	CdS	2.5–8	87% MB removal	[208]
*C. maxima* peel	Iron nZV	10–100	-	[209]
Green tea extracts	Iron NPs		photoactive	[210]
Pomegranate (*P. granatum*)	Iron NPs		Photocatalyst	[211]
*Tamarix aphylla*	Iron NPs		Spherical iron oxide nanoparticle	[211]
*Cynometra ramiflora*	Iron NPs		Photocatalyst	[212]
*K. alvarezii* plant extract	Iron NPs		Dye degradation	[213]
*Peumus boldus*	Silver NPs		18 nm spherical	[214]
*Terminalia catappa* leaf extract	Copper Nps		Tensile strength and thermal stability	[215]
*A. sativum*, *A. cepa*, and *P. crispum*	ZnO NPs		Photoactive	[216]
*Daphne mezereum*	Iron oxide NPs		Photocatalyst	[217]
*A. indica*	Iron oxide NPs		Antibacterial and photocatalyst	[218]
*Cynometra ramiflora*	Iron oxide NPs		Photocatalyst	[219]
*Rhodobacter sphaeroides*	ZnS	6.8	Spherical	[220]
*Rhodopseudomonas palustris*	CdS	8.01	Spherical	[221]
*E. coli*	CdS	2–5	Spherical	[222]
Lemon leaf extract	ZnO-CT		97% Congo Red removal	[223]
*Moringa oleifera* peel	CeO_2_	40	Spherical	[224]
*Prosopis farcta* aerial	CeO_2_	30	Uniformly and spherically shaped	[225]
*Salvia macrosiphon* Boiss. seed	CeO_2_	20–47	Spherical	[226]
Mangrove leaves	TiO_2_	3	Spherical	[227]
*Aspergillus flavus* TFR7	TiO_2_	12–15	Spherical	[228]
Wheat leaf extract	ZnO	12–37	cubic	[229]
*Garcinia xanthochymus* fruit	ZnO	20–30	Spherical shape; spongy cave-like structures; crystalline; hexagonal wurtzite	[230]
*Trianthema portulacastrum*	ZnO	25–90	Crystalline	[231]
*Citrus limon*, *Vitis vinifera*, and *Cucumis sativus* peel	Fe_3_O_4_	8–12	Spherical and polyhedral shape, inverse cubic spinel structure	[232]
*Rubus glaucus* Benth. leaf	Fe_3_O_4_	40–70	Aggregated, spherical	[186]
*Syzygium cumini*	TiO_2_		Spherical, 83% Pb(II) removal	[233]
*Citrus limon*	Fe_3_O_4_		99%, 46% and 48% Pb(II), Cd(II), and As(III) removal	[234]
Mint leaves and orange peels	CuO		Spherical, Qm 89 mg/g and 55 mg/g for Pb(II) and Ni(II)	[235]
*Punica granatum*	NiFe		77% of tetracycline	[236]
*Pseudomonas aeruginosa*	ZrO_2_		Qm 526.32 mg/g of tetracycline	[237]
*Euphorbia polygonifolia*	Fe_3_O_4_@CuO		89%, 94% and 96% metronidazole, ciprofloxacin, and cephalexin removal	[238]
*Abutilon indicum*	MnO		Spherical, 11.5% Cr(IV) removal	[239]
*Aspergillus tubingensis*	Fe_2_O_3_		Spherical, 98.0037% Pb(II), 96.4502% Ni(II), 92.1984% Cu(II), and 93.9913% Zn(II)	[240]

The employment of microbial organisms (bacteria, fungi, yeasts, actinomycetes, viruses, etc.) in nanotechnology and microbial biotechnology is linked to the creation of innovative photocatalysts in a more environmentally friendly manner [171]. Photocatalytic NPs have been widely synthesized using prokaryotic bacterial species. Bacterial synthesis is a suitable alternative for NP manufacturing because of their easy availability in the environment and capacity to adapt to harsh environmental conditions. Bacteria are normally easy to grow and cultivate, and their biosystem may be altered easily [96, 172]. Khan and Fulekar, for example, created NPs of TiO_2_ in the size range of 15.23–87.6 nm by using *Bacillus amyloliquefaciens* bacterial culture. The bacterial species were collected and isolated from effluents of the dairy sector in Mehsana, India. It is worth noting that Fourier-transform infrared (FTIR) spectroscopy revealed the presence of α-amylase, which is primarily involved in the manufacture of TiO_2_ NPs (NPs). Under artificial UV exposure, NPs of biosynthesized TiO_2_ showed photocatalytic degradation of the dye Reactive Red 31 (RR31) [173]. Dhandapani et al. [174] used *Bacillus subtilis* (FJ460362) bacterium to make TiO_2_ NPs with a diameter of 10–30 nm. The formation of an aquatic biofilm was used to test the photocatalytic activity of the produced TiO_2_ NPs. They also discovered that photocatalysis produced H_2_O_2_, which inhibited biofilm formation. *Bacillus licheniformis* microbial strains (MTCC 9555) were used to produce ZnO nanoflowers (200 nm to 1 μm in diameter). Methylene Blue (MB) was used as a model pollutant to test the photodegradation efficiency of the ZnO nanoflowers. Within 60 min, ZnO nanoflowers showed 83% decolorization efficiency. After three recycles, ZnO nanoflowers showed good photostability, according to the authors [175]. Fungi, unlike other microbes, may easily create NPs owing to the presence of enzymes, proteins, and reducing agents on their cell walls. Metal salt solution is quickly reduced in the presence of enzymes on the cell wall, resulting in extracellular NPs. Because of the large-scale production and the ease of processing downstream, this is a financially feasible method. Jain and colleagues demonstrated the production of zinc oxide NPs from zinc salt using *Aspergillus* sp. NJP02, a fungal species. Zinc oxide NPs were produced extracellularly by *Aspergillus* sp. from zinc acetate. UV-induced degradation of the dye Methylene Blue (MB) was demonstrated. The photocatalytic degradation performance of zinc oxide NPs has been reported [176]. The fungus *Trichoderma harzianum* was also utilized to make cadmium sulfide NPs (CdS NPs) in the size range of 3–8 nm with a UV absorption peak at 332 nm. Photocatalytic decomposition of Methylene Blue (MB) dye in a reactor was utilized to test the photocatalytic degradation performance of the biologically generated CdS NPs. After 60 min, with a reaction rate of 0.0076 min^−1^, the degrading efficiency was reported to be 37.15% [177]. Algae are basic photosynthetic, autotrophic organisms that may be unicellular (e.g., Chlorella) or multicellular (e.g., *Chlorella*) (e.g., brown algae). In the same way that yeasts have a limited number of research reports, do does this topic. Algae-based NP preparation is a relatively new field of study [21]. For the production of ZnO NPs, the green microalga *Chlamydomonas reinhardtii* has been used. Fast production of ZnO nanoflowers was described. The photocatalytic activity of these nanoflowers was demonstrated in the presence of sunlight. After 2 h, the photocatalytic effectiveness was found to be 90% [178].

### Green Syntheses of Novel Photocatalysts by Using Plant Extracts

The green synthesis of novel photocatalysts with the help of microorganisms has been studied extensively during the last few decades. The biggest disadvantage of the greener approach to microbial NP synthesis is the maintenance and procurement of microbial strain cultures. Furthermore, proper and careful treatment of human pathogenic bacteria is critical. In this case, any carelessness could result in infection and disease. As a result of its virulence, plant extract-mediated NP synthesis has an advantage over microbial synthesis [179]. Plant-assisted photocatalyst synthesis has been shown to be easy and superior to microorganism-assisted synthesis because it does not require the preservation of microbial cultures. Plant extracts have already been shown to decrease and stabilize a variety of metal cations into stable NPs. Many organic compounds found in plant extracts, such as glucose, fructose, water-soluble hydrocarbons, proteins, and other bioactive substances, can be utilized to reduce and stabilize single and multiple metal cations to NPs in a “one-pot” manufacturing process [120]. Many research groups have already investigated green synthesis pathways for metal NP manufacturing from plant extracts (leaves, flowers, roots, seeds, etc.), highlighting their potential applications [180–182]. Elango et al. also demonstrated that a methanolic extract of *Persea americana* (avocado) seed may be used to make tin oxide (SnO_2_) NPs. Initially, the fabrication of SnO_2_ NPs was confirmed [183].

Ultraviolet–visible (UV–Vis) spectroscopy has also been used in this field. The degradation of phenolsulfonphthalein dye was used to measure the photocatalytic activity of SnO_2_ NPs. The SPR band at 426 nm with a clear surface plasmon resonance was used to determine the appropriate dye degradation period [183]. In another study, root bark extracts of *Catunaregam spinosa* were used to obtain stable spherical tin oxide NPs (SnO_2_ NPs) with an average size of 47 ± 2 nm. The presence of bioactive chemicals in the extract during contact was confirmed by X-ray diffraction (XRD) spectrum analysis and FTIR analysis. The breakdown of Congo Red in a multilamp photoreactor with 92% irradiation was used to measure the photocatalytic activity of the NPs. An initial degradation rate of 0.0952 min^−1^ and pseudo-first-order kinetics were observed in the Congo Red decay [184]. Surendra and Roopan used *Moringa oleifera* peel extract and microwave irradiation to obtain green cerium oxide NPs (CeO_2_ NPs). CeO_2_ NPs were characterized by UV–Vis spectroscopy, XRD analysis, FTIR spectroscopy, and high-resolution transmission electron microscopy (HRTEM). These CeO_2_ NPs also exhibited antibacterial and photocatalytic properties. Gram-negative bacteria (*Escherichia coli*) were more resistant to CeO_2_ NPs than Gram-positive bacteria (*Staphylococcus aureus*). The photodegradation efficiency of CeO_2_ NPs was measured using a Heber multilamp photoreactor with Crystal Violet dye. CeO_2_ NPs were found to have a maximum catalytic effectiveness of 97.5% [185]. Kumar et al. investigated the green synthesis of magnetite NPs (Fe_3_O_4_ NPs) using Andean blackberry leaf extract. XRD analysis, transmission electron microscopy (TEM), FTIR spectroscopy, dynamic light scattering (DLS) measurements, and thermogravimetric (TG) techniques were used to investigate the features of the NPs such as their crystallinity and shape, as well as surface parameters. The NPs were 94% metal and 6% capping ligand, according to the TG analysis. Standard pollutants Congo Red (CR), Methylene Blue (MB), and Methyl Orange (MO) were degraded in the presence of sunlight to test the photoactivity of the Fe_3_O_4_ NPs. The photodegradation of the dyes was also found to be linked to the in situ production of ROS, such as the hydroxyl radical (OH.), superoxide radical (O_2_.), and hydrogen peroxide (H_2_O_2_) [186]. Bishnoi et al. also showed that *Cynometra ramiflora* fruit extract may be used to make magnetic iron oxide NPs (MIO NPs) in a stable manner. The breakdown of MB dye by sunlight irradiation was used to test the photocatalytic activity of the green-produced NPs. Under sunlight illumination, improved production of OH and faster decolorization of MB were achieved thanks to the large surface area of the MIO NPs [187]. Naik et al. employed aqueous *Cinnamomum tamala* leaf extract as a reducing/capping reagent for the manufacture of Au/TiO_2_ nanocomposite through a more environmentally friendly process. The improved nanocomposite showed better degradation of Methyl Orange (MO) dye than Degussa P-25 TiO_2_ under solar irradiation [188]. Rostami-Vartooni et al. [189] examined the synthesis of Ag/TiO_2_ nanocomposite in another study. As a reducing and stabilizing agent, they employed *Carpobrotus acinaciformis* leaf and flower extract. XRD analysis and field emission scanning electron microscopy (FE-SEM) were used to examine the morphology of the Ag/TiO_2_ nanocomposite. The degradation of two distinct dyes was used to measure the photocatalytic activity of the Ag/TiO_2_ nanocomposite. The photocatalytic activity of the Au/TiO_2_ nanocomposite remained constant after four cycles, according to the researchers [189]. *Azadirachta indica* leaf extract, which is rich in bioactive compounds, was shown by Sankar et al. to be useful in the production of titanium dioxide NPs. It was found that the average particle size of these NPs was 124 nm. Under bright sunlight, photocatalytic degradation was carried out, and the photocatalyst demonstrated degradation activity [190]. For the manufacture of titanium dioxide (TiO_2_) NPs, reducing and capping agents can be made from the dried leaves of the plant *Jatropha curcas* L., which are rich in bioactive compounds such as tannins. Under sunlight, the green-produced TiO_2_ NPs were used to photocatalytically reduce real tannery wastewater. The reduction of chemical oxygen demand (COD) and Cr^+6^ was determined to be 82.26% and 76.48%, respectively [191].

Rambutan (*Nephelium lappaceum* L.) fruit extract can also be utilized to biosynthesize stable ZnO NPs with diameters between 25 and 40 nm, according to Karnan and Samuel Selvakumar. The degradation of Methyl Orange (MO) dye under artificial UV irradiation was used to measure the photocatalytic activity of ZnO NPs. The decolorization efficiency was found to be 83.99% after 120 min of illumination. COD values were used to measure the mineralization efficiency, and after 120 min of UV light, significantly lower COD values were observed when applying the ZnO NPs synthesized using biosynthetic methods [192]. Another work used dried *Camellia sinensis* leaf extract to make zinc oxide NPs from zinc nitrate solution via a “one-pot” technique. The degradation efficiency of the produced zinc oxide NPs was higher than that of commercially available zinc oxide NPs [193].

ZnO NPs can also be made from the latex of *Carica papaya* milk (CPM) [194]. Nanoflowers, agglomerated nanobuds, agglomerated prismatic tips, nanobuds, and prismatic tips were among the five nanostructures created. Furthermore, the produced ZnO nanoflowers exhibited superior antibacterial action against *Pseudomonas aeruginosa* and *Staphylococcus aureus*. FTIR spectroscopy, SEM, transmission electron microscopy (TEM), and HRTEM were used to analyze the ZnO nanoflowers. As a result of its small particle size, the photocatalytic activity of the ZnO nanoflowers was increased. The usefulness of the ethanol extract of *Mimosa pudica* leaves has been demonstrated in several investigations, and coffee powder has also been employed to synthesize ZnO NPs. Because of its larger crystallite size, the ZnO NPs made from *Mimosa pudica* leaf extract had lower bandgap energy [195]. Fowsiya et al. [196] recently described a simple method for phytosynthesis of ZnO NPs using *Carissa edulis* fruit extract (*C. edulis*). The production of ZnO NPs is illustrated by the surface plasmon resonance (SPR) at about 358 nm, according to the authors. Decolorization of Congo Red was performed in a photoreactor, showing a most efficient rate constant (*k*) of 0.4947, achieving 97% color removed. *Tabernaemontana divaricata* green leaf extract includes flavonoids, steroids, terpenoids, phenolic acids, phenylpropanoids, and enzymes, according to Raja et al. [197], and aqueous extract was employed for the environmentally friendly manufacture of zinc oxide NPs (ZnO NPs). The antibacterial activity of ZnO NPs was investigated against three bacterial strains: *E. coli*, *Salmonella paratyphi*, and *Staphylococcus aureus*. The antibacterial activity of ZnO NPs against *S. paratyphi* was lower. Methylene Blue (MB) decolorization under sunlight was examined to determine the photocatalytic activity of the generated ZnO NPs, taking 90 min to complete. For greener production of ZnO NPs, *Moringa oleifera* natural extract was employed by Archana et al. [198], and the average particle size was found to be between 100 and 200 nm. These NPs were used to generate hydrogen via photocatalysis. The crystalline structure of ZnO NPs was confirmed by XRD analysis and Raman investigation. Vidya et al. [199] demonstrated green synthesis of zinc oxide (ZnO) NPs utilizing *Artocarpus heterophyllus* leaf (jackfruit) extract. The produced ZnO NPs had a hexagonal wurtzite-like shape with particle size of 15–25 nm, according to TEM investigation. This approach produces ZnO NPs with a high photodegradation efficiency (> 80% in 1 h) against Rose Bengal dye. Langmuir–Hinshelwood kinetics were found to be responsible for the degradation of the Rose Bengal dye.

## Environmental Applications of Photocatalysis

The main applications of photocatalytic phenomena are related to the following processes:

• **Air treatment:** the elimination of ethylene from fruits and vegetables during storage, air stripping of soil for photocatalytic treatment, cleaning of indoor and outdoor air and off-gas emissions, odor elimination, etc.

• **Water treatment:** Digestion and purification of effluent for use in bioreactors and other processes that produce usable water, etc.

• **Active surfaces**: Degradable or antifog materials that can sterilize or sterilize themselves (metals, ceramics and tiles, paints, paper, concrete and cement, glass, textiles, plastics, etc.)

• **Green chemistry:** Photocatalyzed chemical production processes that are more environmentally and economically beneficial

• **Energy conversion:** Water splitting to generate hydrogen gas, or photosynthesis to reduce carbon dioxide gas

### Air and Water Treatment

Photocatalytic reactions require irradiation to begin the reaction. A suitable radiation source is therefore required for photocatalytic reactors; this can be either artificial or natural. A few examples of manmade sources include arc and incandescent lamps, fluorescent lights, lasers, and light-emitting diodes (LEDs). The radiation from these devices can be focused or redirected with the help of reflectors or fiber optics. Reflectors of various types, materials, and concentration ratios can be used to collect solar radiation, or it can be captured directly by the reactor using this technique [241, 242]. It is possible to categorize photoreactor designs for water and air purification based on the radiation characteristics (solar or artificial, concentrated or not), catalyst dispersion (immobilized in various substrates or suspended in solution), reactor geometry (flat plate, parallel plate, U-shaped, fountain, etc.), and operation mode. Many photoreactor designs for water and air purification have been proposed in scientific literature and patents (batch or continuous). Using the kinetic, mass transfer, and radiative transfer equations (RTEs), a simplified or rigorous mathematical model for the photoreactor can be developed following the selection of the photocatalyst and radiation source [243].

Photodegradation rates (*r*) for chemical contaminants in water and gas phases are related to the fraction of covered surface (Eq. 15) according to the Langmuir–Hinshelwood (L–H) kinetic equation (Eq. 15).

15 $$r=-\frac{\mathrm{d}C}{\mathrm{d}t}=\frac{kKC}{1+KC},$$

where *k* is the rate constant, *K* is the pollutant adsorption constant, and *C* is the pollutant concentration. It is generally accepted, however, that the rate constants and orders established using this kinetic model are only apparent. Recent years have seen the development of alternative kinetic models such as the direct–indirect (D–I) model [244], in which the kinetic constants are characterized in terms of the sequence of main events happening during the photocatalytic processes. All of these factors, along with the radiation intensity and wavelength distribution, the type and concentration of pollutants, flow rate, characteristics (turbidity, pH, ionic strength, and dissolved oxygen) of the treated air or water, properties (crystallinity, porosity, doping, loading, etc.) of the catalyst, and reactor design impact on the efficiency and selectivity of the system. The temperature should have little effect on the lamp’s performance because of the photonic activation of the process; however, high temperatures may favor charge carrier recombination and disfavor pollutant adsorption. Each application must first be tested in the laboratory because comparing and scaling up are extremely difficult tasks.

The deployment of effective, cost-effective, and environmentally friendly water treatment technologies is in great demand, fueled by the world’s rising population and more stringent legislation. There is a considerable possibility that heterogeneous photocatalysis, either alone or in conjunction with other processes, could improve current technological options significantly. Considering that microorganisms are the primary source of contamination in drinking water, disinfection is an important photocatalytic technique for this application. Meanwhile, organic materials as well as trace pollutants from medications, insecticides, and personal care products are found in wastewater. Heavy metals or organic compounds may also be present in industrial wastewater. Insecticides such as aldrin, dichlorvos, and lindan (as well as chloroform and carbon tetrachloride, trichloroethylene, and chlorobenzene) and formaldehyde (as well as formaldehyde, phenol, and methylbenzene) are among the most common pollutants. Degradation mechanisms for a few of these have been discovered [245], but the many factors that influence such photocatalytic processes, as well as the lack of standardization in photocatalytic processes, often lead to conflicting conclusions by different researchers.

Continuous flow tubular reactors with TiO_2_ suspensions and nonconcentrating solar compound parabolic collectors (CPC) are used in this experiment [246], and are the most extensively used for aqueous phase photocatalytic reactions. For commercialization, it is necessary to recover the catalyst (e.g., via sedimentation or filtering), although powdered TiO_2_ dispersions are used in most water treatment research studies [247]. The use of titania immobilized on a variety of supports, such as adsorbent substrates, and the design of reactors that maintain high efficiency despite the reduced catalyst surface area and mass transfer limits in immobilized systems necessitate a great deal of effort. Reactors utilizing hybrid membranes and photocatalysis [67] and recoverable magnetic photocatalytic particles are promising solutions [248]. The impact of various operational parameters has already been studied in several books and reviews [67, 245]. The performance of aqueous photocatalytic systems is heavily influenced by the pH of the water, in part because of the low adsorption of contaminants owing to water saturation. The neutral surface charge of TiO_2_ (point of zero charge, PZC) (pH range of 4.5–7.0) results in no interaction with polar substances, such as water.

The low solubility of oxygen (and thus the need for aeration), radiation absorption in turbid waters, or the presence of water natural components that act as scavengers. Additionally, the kinetics and costs, as well as the suitability of this method for water treatment, are governed by reactive species such as carbonates or other inorganic anions [249]. One of the most significant disadvantages of TiO_2_ photocatalysis, as with other advanced oxidation technologies (AOTs), is their relatively high operating costs compared with traditional biological treatments, particularly the slow kinetics compared with the homogeneous photo-Fenton reaction, which makes the latter more appealing to the scientific community despite the chemical consumption. As a result, heterogeneous photocatalytic water purification systems currently focus on the treatment of non-biodegradable wastewater and the use of solar radiation, particularly in remote areas and developing countries, or the combination of other physical or chemical operations to achieve a synergistic effect. Biological treatments, membrane reactors, and physical adsorption do not affect the photocatalytic efficiency, but they do improve the overall process when used in conjunction with ultrasonic irradiation, a photo-Fenton reaction, ozonation, or electrochemical treatment [250, 251]. Since it reacts with water-soluble contaminants that are not biodegradable, photocatalysis is currently a popular pretreatment method before biological water treatment.

### Self-Cleaning Materials

The air-cleaning and self-cleaning, self-sterilizing, and antifogging properties of TiO_2_-containing materials have sparked interest from the scientific community as well as the construction and vehicle sectors. However, these materials are usually optimized for their primary function. Material that is self-cleaning but lacks the adsorption capacity needed for air treatment applications is preferable to material that is smooth, such as the thin coating on a window. The self-cleaning properties of TiO_2_ surfaces can be attributed to a combination of reasons. Adsorption sites for particles such as soot and grime can be removed through photocatalytic elimination of organic deposits as well as the simultaneous inactivation and mineralization of surface microorganisms (self-sterilizing properties). Second, photoinduced superhydrophilicity prevents water from forming surface droplets; instead, when exposed to light, a homogeneous thin water layer covers the surface beneath the dirt, allowing dirt to be washed away readily. Superhydrophilicity also prevents fogging since water attempts to run off the surface (fogging occurs at water contact angles greater than 200°). Metals can be protected against corrosion by using TiO_2_ coatings, which introduce electrons into the metal, or the heat transfer rate can be increased on superhydrophilic surfaces, which reduces water usage while also improving heat transmission. For example, the latent heat of evaporation is employed to cool buildings using falling film evaporators and passive cooling systems based on this effect [252]. Nanofunctionalized thin films over glasses have recently been shown to have antireflective and photocatalytic characteristics compatible to low-refractive-index nanoporous silica and high-refractive-index titania in low-refractive-index nanoporous silica–titania [253].

### Green Materials in Fuel Cells

Fuels produced using green methods or chemistry are a critical source for power generation today. The limited availability of existing fuels, such as petroleum products, results in higher environmental pollution and costs. It is thus critical to find a new source of energy materials that is ecologically benign, low cost, and readily available in Nature. Green photocatalysts enable a process that uses light energy and natural resources to produce fossil fuels. Biomass can be used to produce hydrogen fuels and biodiesel, for example [254, 255]. Hydrogen fuel is one of the zero-carbon transportation fuels that can be made from biomass, but it has limitations in terms of large-scale manufacturing [255]. This type of fuel, which is made from natural resources, can help to reduce greenhouse-gas emissions and enhance the quality of the air we breathe. Nanotechnology is the most effective technique for transferring this approach into the real world [256–258]. This provides a fantastic platform for research organizations to use nanotechnology to develop novel renewable energy materials. Li-ion batteries, flow batteries, supercapacitors, fuel cells, solar cells, and fire- and heat-retardant insulation applications are among the areas where nanotechnology plays an important role in alternative energy production, according to recent studies. Nanotechnology offers a more efficient method of producing solar cells. Essentially, the fabrication process is solely reliant on the absorption of light and the conveyance of charge carriers, both of which are easily accessible and have a low production cost [154]. In dye-sensitized solar cells, coloring compounds taken from plants are utilized for sensitization in dye-sensitized solar cells (DSSCs). The coloring chemicals aid in the absorption of a wider range of light, increasing the efficiency of solar cells. Hydrogen fuels are created by converting biomass, which uses 35% less energy than the electricity required to go 400 km in a battery-powered electric car.

### Green Photocatalytic Disinfection of Water

Water is a necessity for human survival. In our ecology, the availability of fresh, pure water is quite restricted [259]. The majority of water sources have been polluted as a result of urbanization and industry. Textiles, tanneries, and pharmaceuticals are among the businesses that dump waste or byproducts into water bodies. Solar energy is a plentiful natural resource that is freely available on the Earth’s surface, and the collective radiation of sunlight energy can eradicate harmful bacteria in water bodies. Various process characteristics, such as light intensity, incoming light temperature, and pathogen type, can directly inhibit the photocatalytic efficiency of solar disinfection (SODIS) processes [260]. Heterogeneous photocatalysis is the most viable method for effectively killing microorganisms. Clasen et al. [261] reported on a cost-effective strategy for preventing diarrhea in the home. They concluded that the most cost-efficient and effective solar disinfection technology is a household-based water purification system. Even though it is slightly more expensive than chlorination, it has a higher overall disinfection efficacy than SODIS [260]. By immobilizing the photocatalyst under UV irradiation, the water purification process can be carried out in aqueous solution [262]. The disinfection level of the aqueous solution should be measured before and after light exposure at regular intervals. TiO_2_ photocatalysts outperform UVA therapy alone in terms of water disinfection efficiency. Alrousan et al. used two different configurations with and without a photocatalyst (TiO_2_) to demonstrate solar light-induced photocatalytic (SPC-DIS) and solar disinfection (SODIS) of *E. coli*-polluted water [263]. According to recent scientific findings, the most promising strategy for achieving effective catalytic activity under visible light irradiation is doping to achieve metal ion inclusion in the host material [256, 264]. However, research on microorganism disinfection is sparse, and reported publications are likewise few when compared with papers on photocatalytic disinfection of organic pollutants.

### Photoelectrochemical H_2_ Production via Water Splitting

It is widely known that using an appropriate semiconductor photocatalyst can enable effective conversion of solar energy to chemical energy for the generation of clean energy [265]. Thus, using semiconductor photocatalysts with efficient nanostructures that have a high surface-to-volume ratio and a high capacity for light absorption, hydrogen can be created by photoelectrochemical (PEC) water splitting. The two half-reactions that make up a typical PEC cell are (a) the oxygen evolution reaction (OER), which typically takes place on an *n*-type semiconductor as a photoanode, and (b) the hydrogen evolution reaction (HER), which typically takes place on a cathode as a counterelectrode. By evaluating various nanostructured semiconductor photocatalysts, several researchers are making considerable efforts to increase the efficiency of the hydrogen generation rate under solar irradiation [265].

### Photocatalytic CO_2_ Reduction

As of 2019, it was stated that the significant use of fossil fuels caused enormous carbon emissions, with an atmospheric concentration exceeding 400 ppm. A significant quantity of CO_2_ is released into the environment as a result of the excessive use of fossil fuels (e.g., petroleum, gas, and coal) [265]. Around 76% of yearly greenhouse gas (GHG) emissions are attributed to the release of CO_2_, which has serious negative effects on the environment and human health by contributing to climate change, ocean acidification, and ocean warming [265]. By using photocatalytic technology to convert CO_2_ into valuable small-molecule chemical products or energy sources (such as CO, CH_4_, HCOOH, and other chemicals), the energy crisis and these serious ecological problems could be resolved. The conversion of GHGs, such as CO_2_, into useful goods and the reduction of its disastrous release have both been the subject of extensive investigation by several scientists. As a consequence of such development, environmental issues including climatic effects such as ecological degradation, seawater acidification, and the rise in ocean levels could be lessened [265, 266].

### Photocatalytic Dye/Drug Degradation

According to a World Bank assessment, the textile and dyeing sectors are responsible for between 17% and 20% of water contamination [267]. With an annual worldwide output of 8 × 10^5^ tons of dyes, of which around 200,000 tons are textiles and dyes, the textile, leather, food, and paper sectors are principally responsible for manufacturing dye wastewater, according to a newly published report [268]. Numerous synthetic dyes used in textiles, including cationic dyes such as Safranin O, Rhodamine B, Malachite Green, Rhodamine 6G, Methylene Blue, and Crystal Violet, and anionic dyes such as Eosin Y, Eriochrome Black T, Phenol Red, Methylene Orange, and Congo Red, are toxic and harmful organic contaminants that can hinder the photosynthesis process of aquatic plants and pose a threat to the other organic wastes that are damaging to society and the environment are created by the chemical and pharmaceutical industries. To meet the needs of modern lifestyles and expanded healthcare, pharmaceutical and personal care products (PPCPs) have been produced and increasingly used during the course of the last few decades [272]. There are over 3000 commonly used medications, and their use is still increasing globally, according to the European Union market [273]. According to a recent analysis, the amount of antibiotics used globally is estimated to be between 100,000 and 200,000 metric tons. Of the antibiotics used, 70–90% remain chemically unchanged or are eliminated from the body as active metabolites [274]. In addition, the coronavirus disease 2019 (COVID-19) epidemic has considerably expanded PPCP production and usage worldwide in recent years. According to the People’s Republic of China’s National Health Commission, the usage of antiviral and antibiotic medications increased dramatically during the pandemic [275]. Water can contain pharmaceutical pollutants in amounts ranging from ng/L^−1^ to µg/L^−1^. Even at these low concentration levels, they can represent a major hazard to the health of living creatures owing to their chemical and physical characteristics [276]. Numerous attempts have been made to develop highly effective semiconductor-based photocatalysts to photodegrade dye and pharmaceutical pollutants owing to the serious issues associated with dye and pharmaceutical pollutants in water, as well as to the significant advantages of photocatalysis processes in removing harmful pollutants from water.

## Simultaneous Photocatalysis

Significant effort and studies have been devoted to the use of suitable semiconducting photocatalysts in a variety of crucial chemical reactions, e.g., for wastewater treatment, H_2_ generation, CO_2_ reduction, organic transformations, N_2_ photofixation, biomass conversion to valuable products, and heterogeneous photocatalytic reactions, over the past 10 years [265]. These processes are well known in traditional photocatalytic research, and they take place under controlled conditions and are each studied independently in literature. The simultaneous use of two or more functionalities in a single photocatalytic device, however, is a more recent creative strategy [265]. The difficult aspect is that merging two functions into a single photocatalytic system necessitates a novel approach to semiconductor photocatalyst design, control, and engineering with specific properties for each use in a given environment. The groundbreaking study of Kim et al. [277] on simultaneous H_2_ generation and phenolic compound degradation by employing a TiO_2_ surface decorated with platinum nanoparticles and fluorine atoms as a photocatalyst explored this idea of dual-purpose photocatalysis. They achieved full mineralization of organic molecules and found anoxic 4-chlorophenol degradation, which was accompanied by H_2_ generation. Since a suitable photocatalyst characteristic is needed for each operation, conducting two or more types of applications over a single photocatalyst at once is the main problem. As a result, the best approach is to build and employ a unique photocatalyst in at least two distinct concurrent applications. For instance, two-dimensional (2D) semiconductors such as graphene oxide (GO), reduced graphene oxide (rGO), and MXenes and their hybrid combinations can be used to concurrently generate H_2_ and degrade pollutants. Zinc porphyrin metal–organic frameworks noncovalently attached to graphene oxide (SURMOF/GO) were created by Nugmanova et al. [278] in Pickering emulsions, and their photocatalytic activity during the photodegradation of Rhodamine 6G and 1,5-dihydroxynaphthalene was examined. Using methyl viologen as a sacrificial electron acceptor, Nikoloudakis et al. [279] created a covalently connected nickel(II) porphyrin–ruthenium(II) tris(bipyridyl) dyad for a photocatalytic water oxidation process in dimethylformamide (DMF).

## AI-Assisted Photocatalyst Design

The discovery of electrocatalysts [269] and photocatalysts [280] has been revolutionized recently by the development of artificial intelligence (AI) and machine learning (ML) approaches. The term “machine learning” is frequently used to describe processes in which a simulation of the relationship between specified reference or input qualities and the parameters of the output to be predicted from the input is “learned” using a suitable training dataset. A large dataset of 10,560 data points from 584 experiments described in 180 scholarly papers about photoelectrochemical water splitting over *n*-type semiconductors was analyzed by Oral et al. [281] using such machine learning techniques. To establish a relationship between the photocurrent density and 33 descriptors, including the type of electrode, preparation techniques, light irradiation condition, and electrolyte solution, the researchers used a predictive model created by random forest statistics to find patterns in the data. With a root-mean-square error of validation and testing of 0.24 and 0.27, respectively, the obtained bandgap of the electrode was astonishingly excellent. Generally speaking, a more comprehensive training dataset is also needed as an ML model’s complexity rises. Such models may infer catalyst activity without doing real trials or simulating conditions, since they have been trained. Using predictive ML models instead of more traditional experimental or computational approaches might be a cost-effective way to determine the photocatalytic activity of a catalyst on the basis of its parameters. By using this method, the time and resources needed to ascertain the photocatalytic activity may be greatly reduced. Additionally, one method for working with a small quantity of data is to combine domain expertise with data-driven ML model training. Numerous types of photocatalysis domain knowledge are offered from the viewpoint of heterogeneous catalysis, leading to the most recent data-driven machine learning advancements. As a result, the need for tests and simulations decreases through the training of precise prediction models, enabling effective photocatalyst screening. The use of ML to provide trustworthy predictions about the choice of dopants for PEC systems with exceptional performance seems promising. A useful technique for identifying previously obscure links is the examination of correlations between numerous dopant characteristics and the photoelectrochemical performance of doped photoelectrodes [282]. Wang et al. [282] successfully built an ML model that can predict the effects of 17 metal dopants on hematite (Fe_2_O_3_), a typical photoelectrode material. A methodology for analyzing the influence of dopants for their underlying structural properties, as recorded in database S, is provided by Wang et al. [282]. A total of 11 descriptors are included in the database S, including atomic number (N), ionic radius (*r*_i_), atomic radius (*r*_a_), single-molecule bond covalent radius (*r*_c_), chemical valence (Z), M–O bond formation enthalpy based on metal and oxygen, electronegativity (*x*), and melting temperature of pure metal (*T*_m_). Furthermore, utilizing different operational variables as input, scientists have employed a variety of ML techniques to forecast pollution removal using photocatalytic reactions [265].

## Conclusions and Future Perspective Directions

**Fig. 16:**
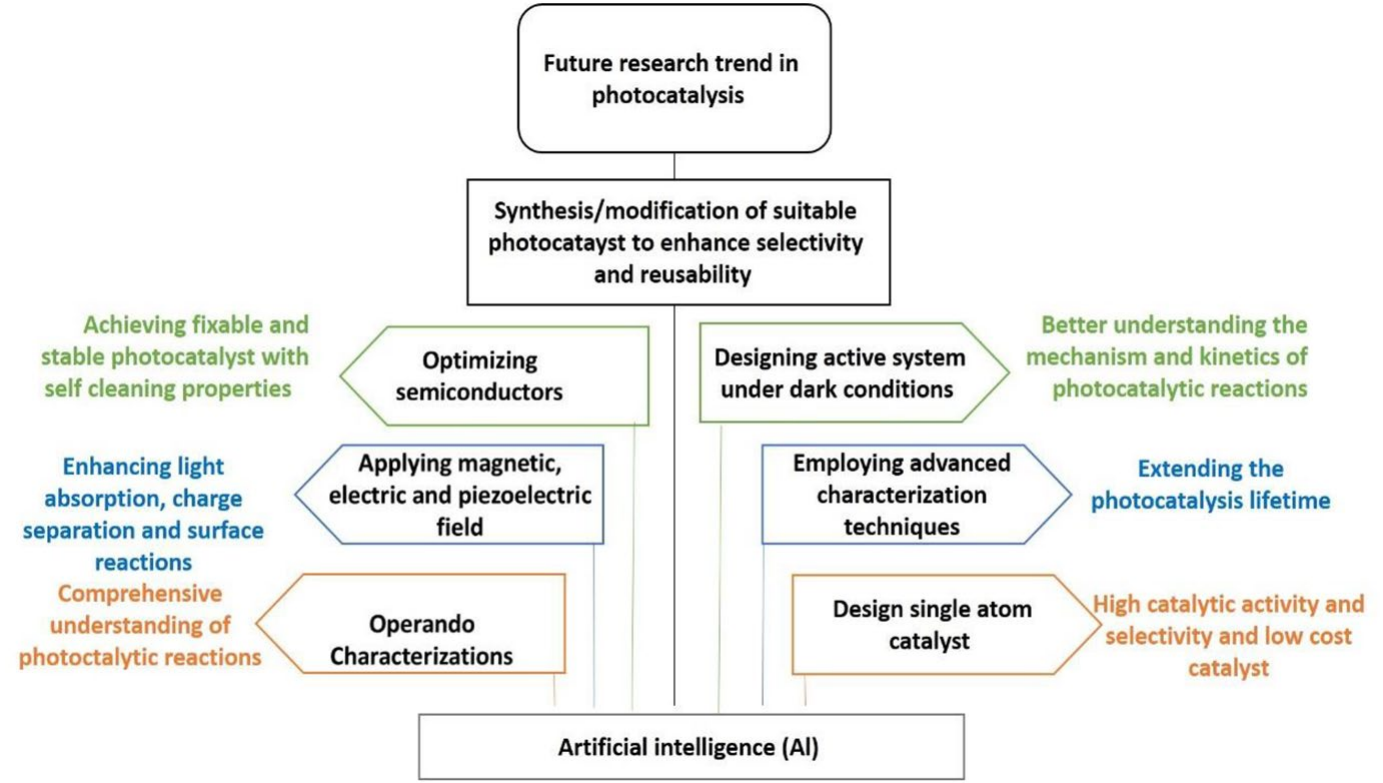
A schematic illustration of future research direction regarding photocatalytic processes [265]

Because of the unique features of nanomaterials, nanotechnology offers the ability to store solar energy and remove organic contaminants from the environment. Artificial photosynthesis systems have a lot of potential. To meet current environmental concerns, green, accessible, and safe techniques for generating such NPs are required. A fast reaction process, low temperature, and limited use of chemicals are all important elements of green or biosynthetic solutions based on biomass feedstock. We can create light-harvesting assemblies, new methods for synthesizing fuels, and instruments to synthesize innovative functional materials for solar cells, water-splitting units, pollution control devices, and more by emulating photoactive green nanomaterials found in Nature. To create metallic NPs without the use of dangerous chemicals, a practical and environmentally friendly approach must be devised. As discussed in this review, researchers around the world are already working on new techniques to make metallic NPs. As a result, the more environmentally friendly production of metallic NPs is gaining a competitive advantage over alternative synthesis methods. This review also covers the use of ecologically friendly synthesis of metallic NPs that also serve as photocatalysts, using a variety of species such as plants, bacteria, fungi, and algae. Even if there has been a lot of progress, the following problems still need to be solved in the future: (1) there is a chance to create new photocatalytic materials with increased effectiveness, selectivity, and reusability by the synthesis of novel materials or modification of existing materials; (2) one novel method for enhancing the photocatalytic performance of semiconductors in a variety of applications is to optimize the semiconductor structure for the creation of flexible and more stable photocatalysts with self-cleaning and flame-resistance qualities; (3) from the economical point of view, it is promising to create photocatalytic systems that are active in the presence of natural sunlight, to significantly increase the photocatalyst lifespan; (4) enhancing photocatalytic reactions by using different types of external field, such as magnetic, electric, and piezoelectric fields, might result in the creation of more effective photocatalysts by improving light absorption, charge separation, and surface reactions; (5) to create more efficient photocatalytic systems for the generation of clean energy and environmental remediation, enhanced characterization investigations might also be carried out to get a better knowledge of the kinetics and processes of the photocatalytic reactions. Designing single-atom catalysts to achieve high catalytic activity and selectivity while lowering practical costs is another important future idea that has gained a lot of media interest lately. The major factor increasing the atomic efficiency of metals in these systems is the isolation of scattered atoms or coordination atoms with surface atoms on a suitable substrate. Last but not least, operando characterization, or simultaneous online examination of photocatalyst performance using in situ imaging techniques such as scanning tunneling microscopy while functioning under actual conditions, aids in gaining a thorough knowledge of the photocatalytic reaction. Future research directions for a photocatalytic process are shown in Fig. 16. As a result, more study is needed to improve present processes and methodologies, which will help the research community and the general public in the future while also posing problems.

## Data Availability

The datasets used in this review are available upon request from the corresponding author of the paper.
